# Predicting suitable habitats for foraging and migration in Eastern Indian Ocean pygmy blue whales from satellite tracking data

**DOI:** 10.1186/s40462-024-00481-x

**Published:** 2024-06-07

**Authors:** Luciana C. Ferreira, Curt Jenner, Micheline Jenner, Vinay Udyawer, Ben Radford, Andrew Davenport, Luciana Moller, Virginia Andrews-Goff, Mike Double, Michele Thums

**Affiliations:** 1 Australian Institute of Marine Science, Indian Ocean Marine Research Centre, University of Western Australia, Crawley, WA Australia; 2 Centre for Whale Research (WA) Inc., Fremantle, WA Australia; 3 Centre for Marine Science and Technology, Curtin University, Bentley, WA Australia; 4 Cetacean Ecology, Behaviour and Evolution Lab, College of Science and Engineering, Flinders University, Bedford Park, SA Australia; 5 Australian Antarctic Division, Department of Climate Change, Energy, the Environment and Water, Kingston, TAS Australia

**Keywords:** Satellite tracking, Movement, Habitat suitability, Species distribution modelling, Threatened species, Machine learning, Spatial prediction, Spatial accuracy, Species management

## Abstract

**Background:**

Accurate predictions of animal occurrence in time and space are crucial for informing and implementing science-based management strategies for threatened species.

**Methods:**

We compiled known, available satellite tracking data for pygmy blue whales in the Eastern Indian Ocean (n = 38), applied movement models to define low (foraging and reproduction) and high (migratory) move persistence underlying location estimates and matched these with environmental data. We then used machine learning models to identify the relationship between whale occurrence and environment, and predict foraging and migration habitat suitability in Australia and Southeast Asia.

**Results:**

Our model predictions were validated by producing spatially varying accuracy metrics. We identified the shelf off the Bonney Coast, Great Australian Bight, and southern Western Australia as well as the slope off the Western Australian coast as suitable habitat for migration, with predicted foraging/reproduction suitable habitat in Southeast Asia region occurring on slope and in deep ocean waters. Suitable foraging habitat occurred primarily on slope and shelf break throughout most of Australia, with use of the continental shelf also occurring, predominanly in South West and Southern Australia. Depth of the water column (bathymetry) was consistently a top predictor of suitable habitat for most regions, however, dynamic environmental variables (sea surface temperature, surface height anomaly) influenced the probability of whale occurrence.

**Conclusions:**

Our results indicate suitable habitat is related to dynamic, localised oceanic processes that may occur at fine temporal scales or seasonally. An increase in the sample size of tagged whales is required to move towards developing more dynamic distribution models at seasonal and monthly temporal scales. Our validation metrics also indicated areas where further data collection is needed to improve model accuracy. This is of particular importance for pygmy blue whale management, since threats (e.g., shipping, underwater noise and artificial structures) from the offshore energy and shipping industries will persist or may increase with the onset of an offshore renewable energy sector in Australia.

**Supplementary Information:**

The online version contains supplementary material available at 10.1186/s40462-024-00481-x.

## Introduction

Accurate data on the occurrence of threatened species in time and space is crucial for managing potential interactions with anthropogenic activities. For many wide-ranging migratory species, satellite telemetry represents one of the most effective methods to collect data to understand species distributions and locations of biological importance (e.g., kernel utilisation distribution, gridded time in area). However, satellite tracking data can be limited in its ability to document potentially important areas across the entire range of animals. This is due to a combination of low sample size of tagged individuals and/or relatively low duration of the deployment of satellite tags due to limitations in battery life, early tag shedding, and/or sensor fouling/failure. As such, the resulting spatial outputs may not be representative of all areas used by the focal species [1–3].

The use of movement data collected by satellite telemetry to predict relative suitable habitat using species distribution models can overcome some of these constraints. Species distribution models identify the statistical relationship between species occurrence and environmental data to derive relative habitat suitability maps [4]. These models were developed for species observation data (e.g., from structured and opportunistic surveys), but for many marine migratory species, observational data is often limited in space and time, and often only represents a small proportion of biologically important activities exhibited by individuals. In contrast, satellite telemetry enables the semi-continuous collection of data, not restricted by observational limitations, and the ability to estimate specific behaviours (e.g., migratory, area-restricted movements). In recent times, this method has been widely applied in the marine environment to define distributions of rare or under-sampled species [5], assess range shifts from climate change [6, 7] and for supporting and assessing the delineation of protected areas [8]. The use of satellite tracking data in species distribution models has been limited historically because the data represents presence-only and is autocorrelated requiring application of complex techniques that can handle these datasets [9]. However, the definition of suitable habitat from satellite telemetry data allows for further inferences of species behaviour (e.g., foraging, migration, resting) in relation to the predicted relative habitat preference, thus providing additional decision support for managing threatened and poorly understood species in habitats of interest [4, 10].

The Eastern Indian Ocean stock of pygmy blue whales (*Balaenoptera musculus brevicauda*) is one of two recognised blue whale (*Balaenoptera musculus*) subspecies in the Southern Hemisphere (henceforth simply referred to as ‘pygmy blue whale’). The pygmy blue whale has currently not been evaluated under the International Union for Conservation of Nature (IUCN) Red List due to the paucity of data on distribution and population trends [11]. The Australian populations of blue whales have, however, been evaluated under the Australian Environment Protection and Biodiversity Conservation (EPBC) Act as ‘Endangered’. The pygmy blue whale migrates from austral summer feeding areas in the Subtropical Convergence Zone (40° S to 55° S) and in south Australian waters towards the equatorial region in the Banda Sea [12]. Recognised aggregations for feeding (predominantly on krill) along their migratory route occur in the Perth Canyon in the southwest of Western Australia (WA), and the Great Southern Australian Coastal Upwelling System in southern Australia [12–15]. Their migration route passes through areas subject to fisheries, offshore oil and gas activities, shipping routes and areas proposed for offshore renewable energy developments [12, 16–18], exposing them to a wide range of threats.

In the Eastern North Pacific, where blue whales are at risk of ship strike, satellite tracking data (combined with observation data) was used to develop a dynamic predictive model of blue whale distribution off California (known as *WhaleWatch*). Seasonal predictions of the probability of occurrence of blue whales from this model allow marine user groups such as the shipping, offshore energy and fishing industries to consider spatial and temporal adjustments to their activities to limit interactions with this threatened species [10]. Given this success, we set out to try a similar approach in Australia, where mining for oil and gas (offshore) and iron ore (on land) has resulted in the development of large ports and an increase in shipping and vessel movement, exposing whales to threats such as noise interference, vessel disturbance and collisions [17]. In order to accurately assess impacts and develop mitigation strategies, an understanding of pygmy blue whale habitat use is needed. In particular, a management strategy that takes into consideration the dynamic nature of the drivers of pygmy blue whale foraging and that these animals appear to feed during migration [12, 19, 20].

Here, we combine existing satellite tracking data from across most of the known range of this subspecies with targeted new deployments of satellite tags to increase the sample size of tracked whales. We use this dataset to model the relationship between whale presence and remotely sensed environmental variables to identify their relative probability of occurrence and define relative habitat suitability across their distribution. In addition, we use movement models to determine where pygmy blue whales displayed low move persistence indicating foraging, resting and/or reproductive behaviours, as opposed to migratory movements, to develop separate habitat suitability models for foraging, migration, and foraging/reproduction areas. We also attempt to predict relative habitat suitability into areas of interest, sometimes beyond the geographic location of training data. However, to assess uncertainty in the predictions and to aid their applicability to management, we implement evaluation metrics such as spatial Kappa and spatial AUC [21] to map the both accuracy and uncertainty of our spatial predictions. Although our sample size is still relatively low compared to other predictive modelling approaches like *WhaleWatch* [10] (n = 38 vs 104), we develop a workflow where additional data can be incorporated in the future, as the dataset grows.

## Material and methods

### Satellite tracking data

Satellite tracking data for pygmy blue whales tagged along the western and southern Australian coasts were compiled for the analyses. This included existing (n = 9 from Perth Canyon, WA, [12]; n = 13 from Bonney Coast, [14]; n = 6 from Ningaloo and n = 4 from Perth Canyon, [18]) and new data (unpublished) from tag deployments in the Perth Canyon, Western Australia in 2022 (n = 6) (Table S1, Figure S1). Please see the cited papers for tag deployment methods. More recent tag deployments were conducted using Wildlife Computers LIMPET (Low Impact Minimally Percutaneous Electronic Transmitters, type: SPLASH10-F-333) containing GPS receivers, thus providing both Argos and GPS location data (Table S1). Older deployments from 2009, 2011 and 2015 [12, 14] employed implantable Wildlife Computers Satellite Position Only Tags (SPOT), providing Argos location data only (Table S1). Argos locations are assigned a location class (LC) by CLS Argos based on the error associated with the location estimate. The spatial error (95th error percentiles) for these location classes (3, 2, 1, 0, A, B, Z) have been independently assessed as 1.5, 3.3, 7.6, 36, 60, 163 and 220 km respectively for unfiltered data [22, 23]. For GPS locations, location fixes obtained from six or more satellites are associated with much smaller errors of around 10 s of meters [24].

A state-space model (SSM) [25, 26] was applied to the raw Argos and GPS location data combined using the R package *foieGras* [27] (now named *aniMotum*; [28]) to account for location error and autocorrelation between locations along the track. To ensure the model did not overfit, we selected the time step for each predicted track to be in-line with the average number of actual locations (both Argos and GPS) received by the satellite tag per day. Tracks with large gaps (> 5 days) were split and each portion of data analysed separately. All models were checked for convergence.

**Fig. 1:**
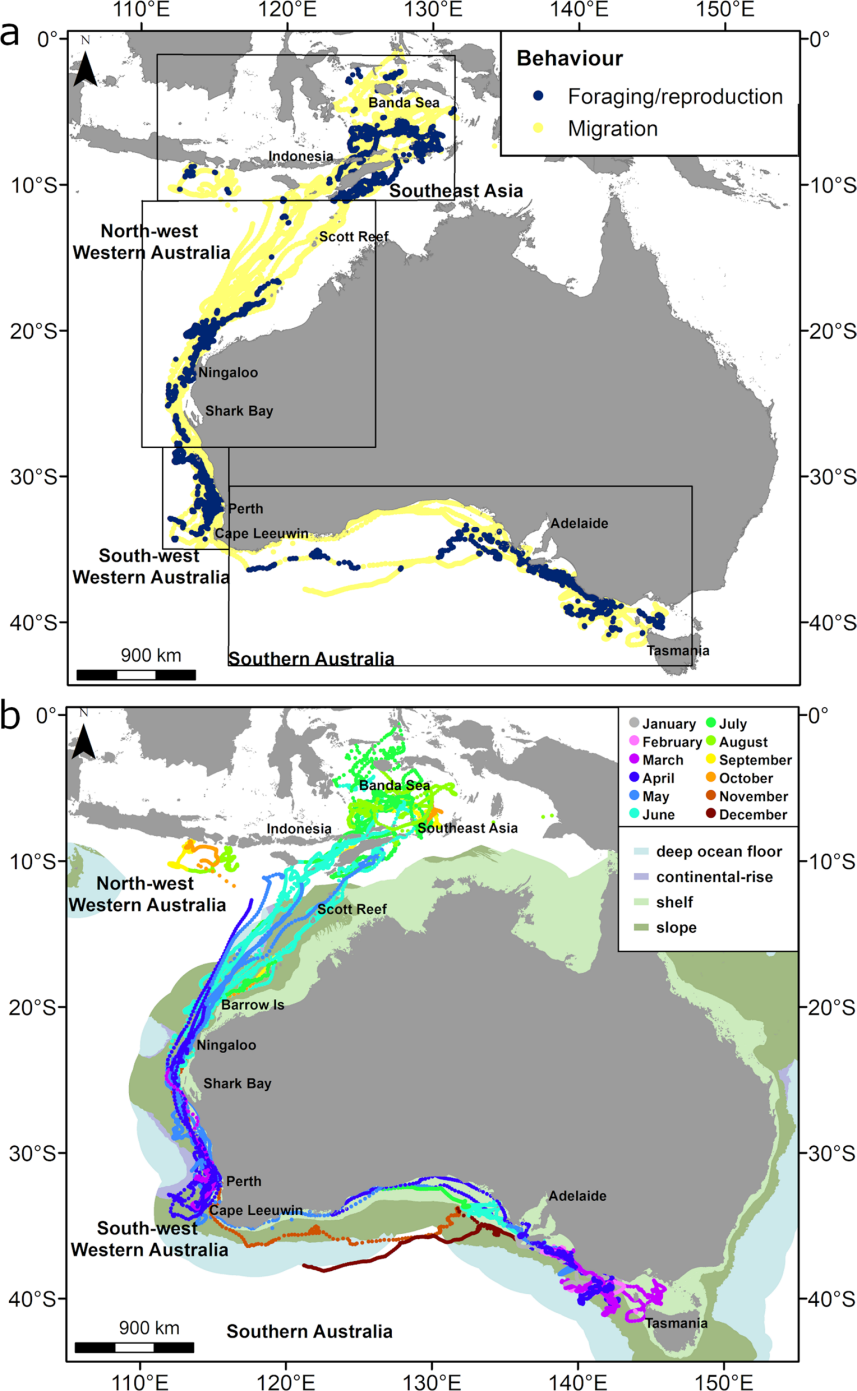
State-space modelled (SSM) satellite tracks of 38 pygmy blue whales and the boundaries of the four regions for which habitat modelling was undertaken. **a** SSM tracks with each location classified as low (g < 0.8, blue; indicative of foraging and/or reproduction) or high (g > 0.8, yellow; indicative of migration) move persistence (g) and **b** SSM satellite tracks with locations colour coded by month of the year, overlayed with geomorphologic features [32]. Note that the North West and South West data were combined to model high move persistence (migration) in Western Australia

A move persistence model was then implemented on SSM tracks, so that a move persistence value (*g*) was calculated for each location [27, 29]. Move persistence (*g*) is an index ranging between 0 (decrease in speed and directionality) and 1 (increase in speed and directionality) [29]. Relatively low move persistence is indicative of ‘area restricted search’ behaviour such as foraging, but can also indicate resting and reproduction, whereas relatively high move persistence generally represents behaviours such as transiting and migration. Based on Thums [18], locations with move persistence (*g*) < 0.8 were classified as behaviours linked to foraging or reproduction and locations with move persistence (*g*) ≥ 0.8 were classified as migration (Fig. 1), with separate habitat models developed for each behaviour. We further split low move persistence locations as those occurring within Australian waters, which we label as foraging (although whales may also be resting while in these areas), and those occurring in the Southeast Asia region which we label as “foraging/reproduction” (although whales may also be resting). We did this based on the view that low move persistence in the latter region is thought (though not confirmed) to be related to reproduction (calving/breeding) but foraging likely also occurs [30], whereas low move persistence in Australia is thought to be related to foraging predominantly [12, 31].

### Environmental variables

Environmental variables most commonly used to model blue whale movement and habitat [10, 14, 18, 30, 33–35], and other marine megafauna [36] were considered as covariates to model habitat suitability for pygmy blue whales. However, not all environmental variables were available with appropriate spatial and temporal resolution for the whole pygmy blue whale distribution. Thus, we selected only those that had spatial resolution < 20 km (Table 1) to match grid cell sizes suitable for modelling satellite tracking data to define distribution and identify important areas within regional extents [1]. Bathymetry data were obtained from the General Bathymetry Chart of the Oceans Gebco15 database in a 30 arc-second resolution grid (http://www.gebco.net). Rugosity was calculated from the bathymetry data in ArcGis 10.8 using the Benthic Terrain Modeler tool as a measure of benthic habitat heterogeneity. Distance to closest canyon was calculated from the edge of each canyon feature using a layer of mapped geomorphic features of the Australian margin [32]. Remote-sensed data used were 5-day and 8-day means and monthly standard deviations rather than daily values to reduce the occurrence of large areas of no data due to cloud cover, which would result in missing data across the model domain. We included measures of chlorophyll*-a* taken 14 days prior to the whale being present at a location to account for the temporal lag that exists between primary production (chlorophyll*-a*) and zooplankton (krill) abundance [37], although time lags from primary production vary across ecosystems and season and can range from a few days to months [38]. We used sea surface height anomaly, which is a measure of the difference between long-term average sea-level for a regional ocean and the measure observed by a satellite at a point in time, as indicative of the presence of upwelling and downwelling of water that might affect productivity and/or be related to the presence of eddies that can aggregate zooplankton [39]. We also included the predictor Month in our models to account for the seasonality of the movement behaviour of pygmy blue whales (Table 1). We used the layers of geomorphology of the Australian margin and adjacent seafloor [32] and [40] to overlay predicted suitable habitat over shelf, slope, and ocean basin bathymetric features within Australian waters and the Southeast Asia region, respectively, and calculated the percentage of suitable habitat within each bathymetric feature.

**Table 1:**
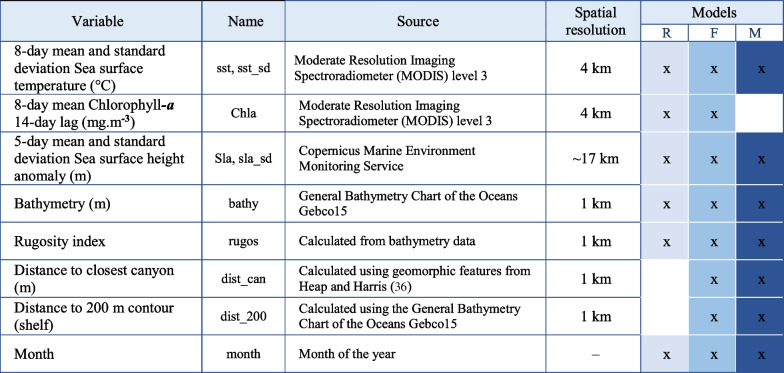
Environmental variables used as covariates to model pygmy blue whale habitat suitability

Shaded cells indicate which variables were considered in the model for each behaviour and cells without shading indicate variables not considered for each behaviour model. R = Foraging/Reproduction (low move persistence in Southeast Asia), F = Foraging (low move persistence in Australian waters), M = Migration (high move persistence)

We assessed correlations between environmental variables prior to the analysis by performing a pairwise Pearson’s test to remove highly collinear variables (< − 0.6 or > 0.6; [41]) keeping the variable that had higher importance score in preliminary testing. Although correlations between variables do not affect the modelling framework gradient boosted models [42] used here (see below), they can influence variable importance scores and partial dependence plots [43].

### Habitat model

We developed a use-availability (or case control) design to estimate relative probability of occurrence, and thus relative habitat suitability, using presence-only species occurrence data satellite tracking data [2, 3, 44]. We modelled relative habitat suitability separately for foraging (low move persistence in Australian waters), foraging/reproduction (low move persistence in Southeast Asia), and migratory (high move persistence in Australia) movement behaviours, because pygmy blue whale habitat and environmental requirements likely differ during these behavioural modes [45]. For the foraging relative habitat suitability models we split the Australian pygmy blue whale range into three regions to account for the different environmental setting in each area [18]: North West Western Australia; between 11.5° S and 28.8° S from Ashmore Reef to Shark Bay, South West Western Australia; between 23.6° S and 32° S, from Cape Naturaliste to Shark Bay, and Southern Australia; as the area of the coast with latitudes greater than 32° S and longitude greater than 116° E from Cape Naturaliste to Tasmania (Fig. 1a). For migration models we used the Australian section of the tracks (42° S to 11.5° S) only as although transit movement exists in Southeast Asia, these were short sections of track and were not considered as part of their seasonal migratory behaviour. We also split the Australian migration data between Southern Australia (area of the coast with latitudes greater than 32° S and longitude greater than 116° E from Cape Naturaliste to Tasmania) and Western Australia (latitudes between 32° S and 11.5° S, which combines the South West and North West regions), to account for the different environmental setting in each area (Fig. 1a).

Gradient boosted models (GBMs) were used to model relative probability of occurrence for pygmy blue whales using the packages *gbm* [46] and *caret* [47] in R [48]. Gradient boosted models sequentially add regression trees to an initial weak tree, incrementally correcting predictions based on the error learned by earlier trees to minimise loss of predictive performance or deviance explained by the model [42, 49]. We selected gradient boosted models over generalised additive models due to their better performance with larger datasets and due to their ability to fit both linear and non-linear responses (i.e., higher values for accuracy metrics; see Supplementary Material 1). This modelling framework has shown generally higher explanatory power for modelling blue whale habitat models [9], and to describe the relationship between blue whale distribution, prey and environment [50, 51]. Additionally, this method is well-suited to model complex and non-linear ecological relationships, and minimises the effect of temporal autocorrelation [42]. We modelled the response variable presence and pseudo-absence using a Bernoulli error distribution, with the locations from the SSM tracks representing pygmy blue whale presence information. We used the term ‘pseudo-absence’ as it is not possible to obtain true absences (e.g., locations where the whale could have gone but did not) from tracking data [9, 52]. Consequently, pseudo-absences were simulated based on movement metrics extracted from the real tracking data (Figure S2). The simulation was done for each migration, foraging and foraging/reproduction region separately. Hence, simulated locations were given the same behaviour classification as the original track.

Each pseudo-track started at the first position of the actual track they were based on. From there each location was created semi- randomly, that is, they were constrained by the duration, and the distribution of step lengths and turning angles from the actual track [9, 52]. To further reduce the randomness of simulated tracks, we oriented each simulated location to the end point (defined by the last location estimate received from the tag). We also included an 'offset' parameter that added a randomised offset to the turning angle at each step and helps prevent the simulated tracks getting stuck inside coastal bays. Offset values used were either 180° or 300° with simulated tracks inspected visually to determine convergence, by ensuring the track did not get stuck inside bays or deviate outside model boundaries (Fig. 1a). Five simulated tracks were generated for each actual track so that the model would have five pseudo-absence data points for each presence location. All data points (presence and pseudo-absences) were then bounded by a regional model extent for each region (Fig. 1a) to avoid outliers and erroneous extrapolation of model predictions.

Environmental variables (Table 1) were extracted for all locations from actual tracks (presence) and simulated tracks (pseudo-absences) matching the date-time information (as 5-day or 8-day averages) of locations and pseudo-absences (Tables S1, S2). Some environmental covariates (chlorophyll*-a*) were log transformed to stabilise variance and ensure better model fitting.

#### Model selection and evaluation

Model settings (interaction depth, number of trees, minimum node size, shrinkage rate and bagging fraction) were optimised for each model (each behaviour and region separately) by performing a grid tuning routine over every possible combination of the model settings parameters. The final model included the settings selected by the lowest relative mean squared error and this model was then used to analyse the relationship between presence of whales and environment variables. We withheld a random sample of 25% of the data (using all tracks combined for each region and each behaviour) to use for model performance estimates (testing set) and used 75% (training set) for each analysis. We used the Spearman correlation coefficient to assess accuracy of the model by comparing model fit values (predicted set) against the testing set, with correlation values > 0.5 considered to be suitable (with higher values indicating stronger correlation and thus better fit). Variable importance was assessed using the relative influence of reducing error rate, thus adding predictive accuracy [53].

Predictions from gradient boosted models were used to provide a spatial representation of habitat suitability based on the relationship between pygmy blue whale presence and environmental variables included in the final model. Model predictions ranged from 0 (unsuitable) to 1 (suitable habitat) and indicate increasing relative probability of occurrence, and thus relative habitat suitability.

Spatial predictions were made against the environmental variables included in the final model. For predictions, due to limitations on sample size (number of tracks, temporal and spatial resolution of data; See Discussion for more details) an averaged raster of each environmental variable was created across the temporal extent of the whale tracking data in each modelled region and for each behaviour (Fig. 1b, Table S2). Available raw environmental data also had different spatial resolutions (Table 1), hence, prior to prediction, average environmental data grids were resampled to create new raster grids that matched the coarsest resolution of all variables used in each of the final GBM models for each region and behaviour.

To identify the cut-off between ‘suitable’ and ‘unsuitable’ habitats from model predictions, we applied a threshold. Thresholds are often applied to habitat suitability model outputs to transform continuous outputs of relative probability of occurrence (from 0 to 1) into binary maps (0 = ‘unsuitable’, 1 = ‘suitable’), aiding in interpretation and applicability for management [54–57]. We applied a conservative threshold method to our predictions (in comparison to the traditional default of 0.5 as the cut-off) by estimating the threshold value for each model where the predicted prevalence (proportion of locations that are occupied) is equal to the observed prevalence [56] to identify suitable habitats for pygmy blue whales [58].

#### Spatial prediction validation

Machine learning algorithms such as gradient boosted models are powerful tools in spatial mapping and predictive modelling as these models can fit complex relationships. However, when these algorithms are used to predict distribution in areas outside the spatial extent of the training data, it is important to assess the confidence in these predictions; i.e., how accurately can the model predict occurrence in areas with environmental parameters that it may have never “seen” and thus, identify and map uncertainty [59, 60]. Providing an assessment of accuracy and uncertainty is key to ensure the resulting habitat suitability maps are interpreted correctly, particularly for management decisions.

Model accuracy was firstly evaluated by the area under the receiver operating curve (AUC; Fourcade et al. 2018) to assess the ability of the model to discriminate between presence and pseudo-absence points, and also by Kappa to measure the expected agreement between the prediction and actual presence data [61, 62]. These metrics provide an accuracy value estimated across the entire prediction area and were termed ‘global’ model accuracy estimates. The AUC values range between 0 and 1, with AUC values considered excellent for values between 0.9–1, good between 0.8–0.9, acceptable between 0.7–0.8, moderate between 0.6–0.7 and poor for AUC values below 0.5 [63]. Kappa values range from − 1 to 1 with results considered to indicate excellent agreement for values 0.8–1, substantial for values 0.6–0.8, moderate for 0.4–0.6, fair for 0.2–0.4, slight for 0–0.3 and poor for values ≤ 0 [64]. If a model resulted in low global accuracy values, we identified the explanatory variables with the lowest relative influence in the model and those that showed large gaps of no data in its spatial distribution and removed them from the training dataset. The model was then run again without such variables until it reached acceptable validation metrics (global AUC > 0.6). Conditional plots for all variables included in the final models were plotted to indicate the relationship between pygmy blue whale occurrence and environmental variables.

To spatially validate our model predictions, we calculated spatially varying estimates of accuracy [59]. To do this, we used a spatially varying version of Kappa and AUC (termed ‘spatial Kappa’ and ‘spatial AUC’) (Comber et al., 2017). Although Kappa is widely used because it can account for ‘by chance’ agreement in models, it can have some limitations in relation to unbalanced sampling and variation in data density [62] which may be relevent for tracking data. Thus, we also present the spatially varying values of AUC. While it does not account for ‘by chance’ agreement in the same way as Kappa, this metric is not effected by unbalanced sample issues in the same way, and provides a spatial estimate of model discrimination to aid interpretation of the spatial predictions. Spatial Kappa and spatial AUC were calculated using a moving window spatial kernel [21, 65]. We used the variogram (range and slope) of the spatial correlation of the data (presence and pseudo-absence) to determine the size of the moving window spatial kernel [66]. Where variogram slopes were greater than 0.8, we used a kernel size of two times the mean variogram range of spatial correlation of the data, and a kernel centroid equal to the mean variogram range. When variogram slopes were less than 0.8, we used a kernel size of 0.5 of the mean variogram range of spatial correlation of the data and, a kernel centroid 0.25 times the mean variogram range. We plotted spatial Kappa and spatial AUC values to assess how predictive accuracy varied spatially and used the moderate values of spatial Kappa and spatial AUC (> 0.4) as an accuracy threshold above which prediction was considered validated.

## Results

A total of 38 tracks were included in our analysis, with 19 tags deployed in the Perth Canyon, Western Australia, 13 in the Bonney Coast Upwelling, South Australia, and six offshore of Ningaloo Reef, Western Australia (Table S1). Median track duration was 49 days (ranging from 8 to 384 days). For whales tagged off the Bonney Coast, tracks had a median duration of 60 days (3–384 days). Tags deployed in the Perth Canyon, had median duration of 28 days (7–141 days) and for Ningaloo Reef this was 52 days (14 to 122 days). For all tracks combined, 33% of locations were classified as foraging and foraging/reproduction (g < 0.8) and 67% as migration (g ≥ 0.8) (Fig. 1a).

Pygmy blue whales were tagged prior to the start of or during their northbound migration, and moved progressively along the coast from tagging sites at foraging areas (Table S1, Fig. 1) towards their presumed breeding/calving area in the Banda Sea region [12] (Fig. 1b). Whales tagged in the Bonney Coast started moving west towards Cape Leeuwin between April and May, although some whales were still displaying low move persistence (presumed foraging behaviour) within Southern Australia and tags ceased transmission before they reached the South West of Western Australia (Fig. 1b). Once whales reached Cape Leeuwin, they shifted their movement to a northward direction. Similarly, whales tagged in the Perth Canyon in April and May progressively moved north along the coast of Western Australia reaching Ningaloo around May–June (Fig. 1b). After that, tracks sprayed out with some whales crossing the abyssal plain and others following the shelf break, reaching Indonesia and Banda Sea in June–July, where they stayed until September–October (Fig. 1b). Tag deployments from only two of the whales provided data on the return trip (Figure S3). These whales started moving south in September–October making their way down the North-West coast of Australia, with one of the whales reaching Ningaloo Reef before transmissions ceased. The other whale continued to move south along the Western Australian shelf edge, arriving in Southern Australia in November and reaching the western edge of the Bonney Upwelling in December when the tag stopped transmitting (Figure S3).

Collinearity between predictor variables was relatively low (Figure S4) and within the accepted range (from − 0.6 to 0.6). Collinearity between distance to the 200 m bathymetry contour (*dist_200*), a proxy for the shelf break, and distance to canyon (*dist_can*) (Table 1) was the only pair above (0.86) the cut-off. The variable *dist_200* was also moderately collinear to bathymetry (Figure S4); therefore, it was the one selected to be excluded from the analysis.

### Foraging

#### North West Western Australia

**Fig. 2:**
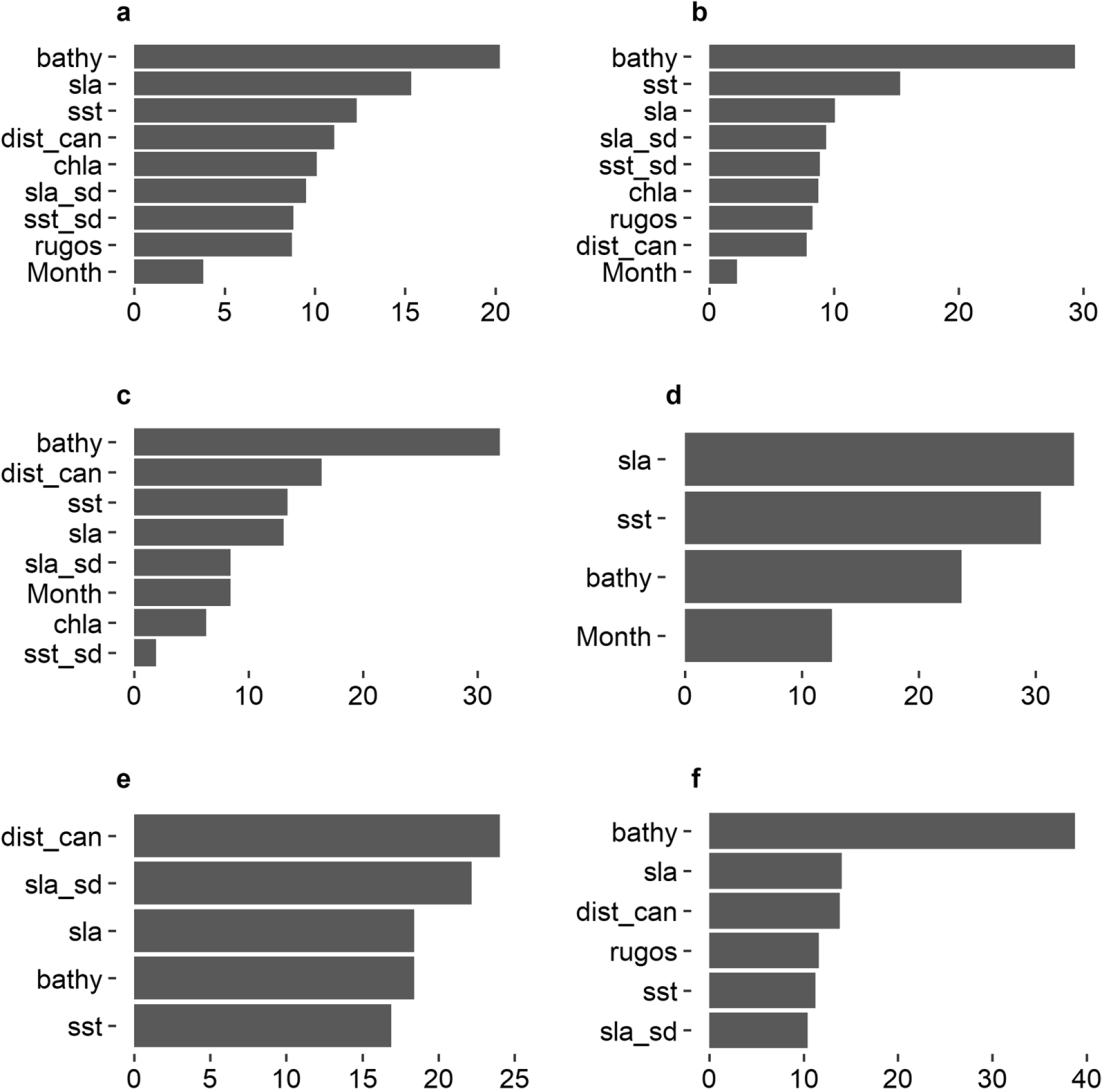
Variable importance plots for gradient boosted models using presence/pseudo-absence of satellite tracked whales displaying low move persistence (presumed foraging movement behaviour) in North West WA (**a**), South West WA (**b**) and southern Australia (**c**); displaying low move persistence in Southeast Asia (presumed foraging/reproduction area) (**d**); and during high move persistence (migration movement behaviour) off western Australia (**e**) and Southern Australia (**f**). Variable importance is presented in relation to their contribution to reducing error rate, where the higher the value the more important the variable. Variable names are described in Table 1

This subset of the dataset included presence and pseudo-absence from 17 pygmy blue whales that displayed foraging movement behaviour in North West WA (Fig. 1, Figures S2, S3). The gradient boosted model had a global AUC of 0.85 and global Kappa of 0.36, and a correlation between prediction and testing datasets of 0.53, indicating the model had a moderate to good performance. The final model included all variables tested for foraging (Table 1, Fig. 2a, Supplementary Material 1).

**Fig. 3:**
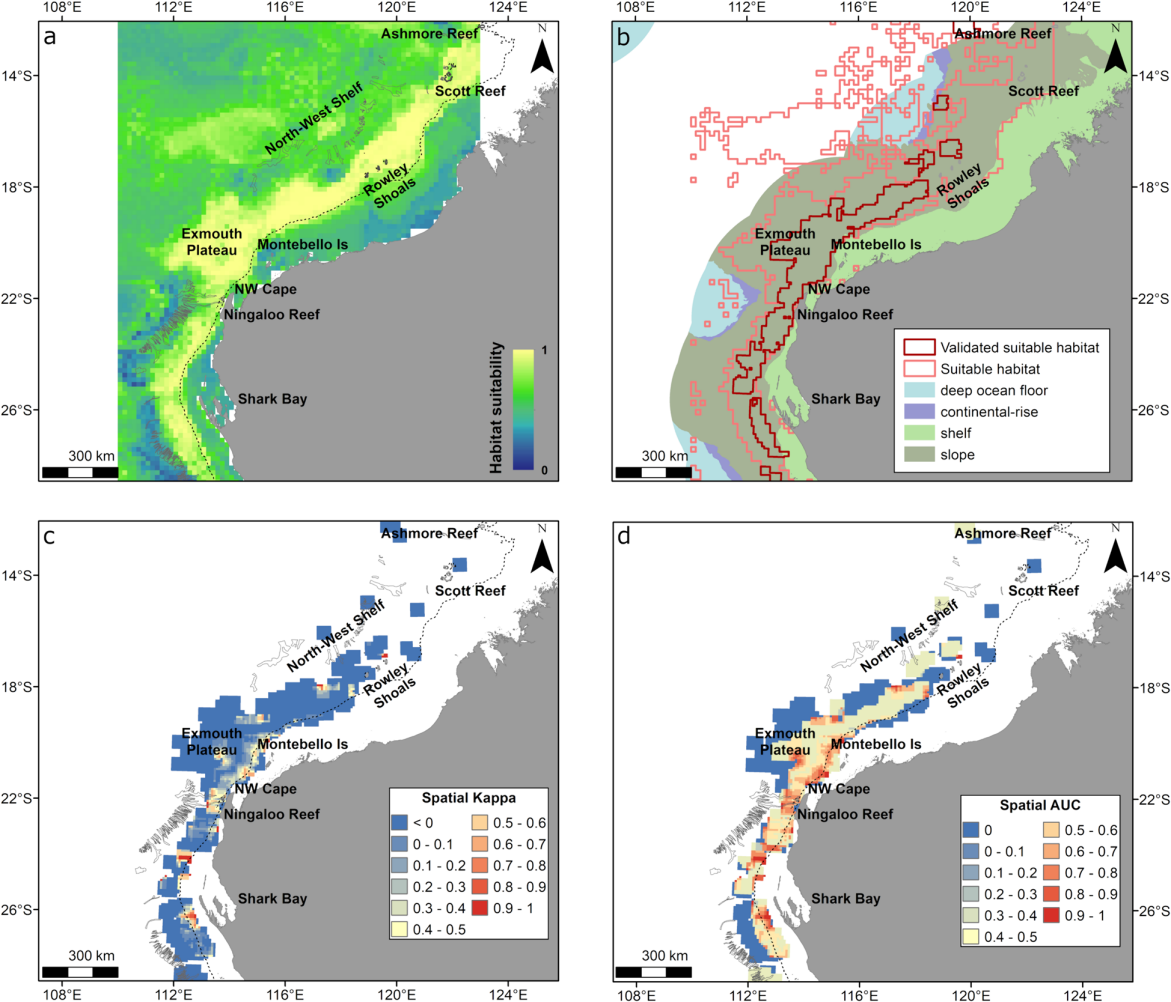
Habitat suitability predicted from the gradient boosted model and spatial accuracy maps for whales displaying foraging behaviour in the North West of Western Australia (April to September). Shown are **a** continuous (0–1) relative habitat suitability, **b** thresholded suitable habitat (pink) and validated suitable habitat (red; suitable habitat restricted to areas with spatial AUC and spatial Kappa > 0.4) overlaid with geomorphologic features [32], **c** spatial distribution of model accuracy (spatial Kappa) and **d** spatial distribution of model accuracy (spatial AUC). Black dotted contours represent the 200 m bathymetry (shelf break), and canyons are outlined in light grey

Spatial predictions of whale relative probability of occurrence in relation to all environmental variables included in the model showed high habitat suitability extending out from the 200 m bathymetry contour (shelf break) in the North West of Western Australia (Fig. 3a, b). The highest relative suitability occurred from just south of Shark Bay to Scott Reef along the shelf break (200 m bathymetry contour) and slope, also extending to the Exmouth Plateau (Fig. 3a). Suitable foraging habitat was identified as a large semi-continuous area from the southern extent (28° S) to the northeastern edge of the modelled region (11.5° S) (Fig. 3b). Using the geomorphology layer [32, 40], suitable habitat occurred almost exclusively on slope (91% of suitable habitat), with a small amount of suitable habitat in deep ocean floor (7%) and minimal suitable habitat on the shelf (2%) (Fig. 3b). However, spatial Kappa and spatial AUC values had a large variation indicating that the model performed better at some areas than others (Fig. 3c, d). Areas along the shelf break and slope between the southern extent of the modelled region and Rowley Shoals had reasonable performance (spatial AUC and spatial Kappa > 0.4), including areas on the slope off Rowley Shoals, validating suitable habitat in that area (Fig. 3b). Spatial Kappa and spatial AUC scores were low, indicating poor predictive performance, between Rowley Shoals and Scott Reef (both < 0.2), at Ashmore Reef and in some areas beyond the shelf break (Fig. 3c, d).

#### South West Western Australia

This subset of the dataset included presence and pseudo-absence data from 20 pygmy blue whales that displayed foraging behaviour in South West Western Australia (Fig. 1, Figures S2, S3). The gradient boosted model had a global AUC score of 0.86, a global Kappa of 0.50 and a correlation between prediction and testing dataset of 0.59, indicating a reasonable fit. The final model included all the variables tested for foraging (Table 1, Fig. 2b, Supplementary Material 1).

**Fig. 4:**
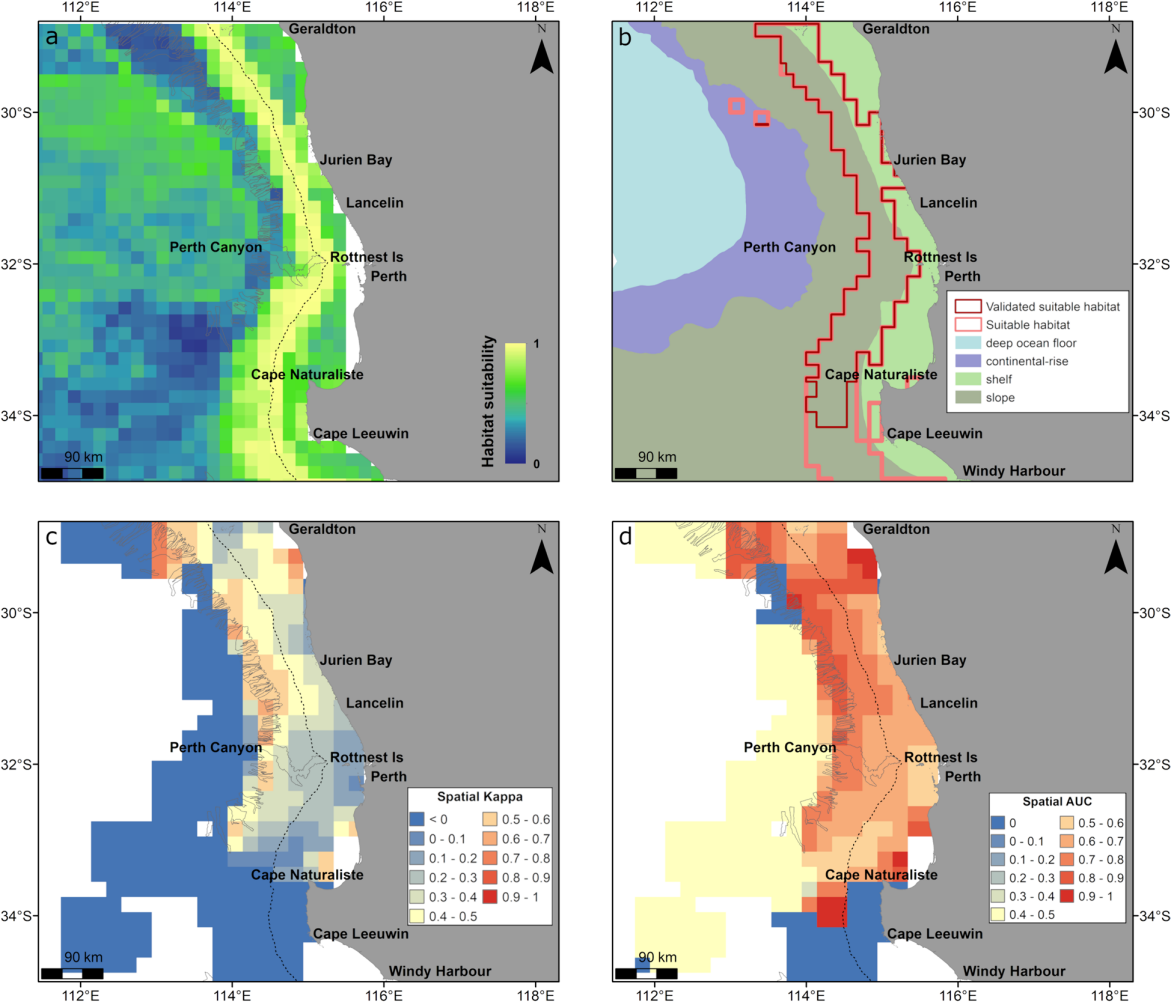
Habitat suitability predicted from the gradient boosted model and spatial accuracy maps for whales displaying foraging behaviour in the South West of Western Australia (March–June, November). Shown is **a** continuous (0–1) relative habitat suitability, **b** thresholded suitable habitat (pink) and validated suitable habitat (red, suitable habitat restricted to areas with spatial AUC and spatial Kappa > 0.4) overlaid with geomorphologic features [32], **c** spatial distribution of model accuracy (spatial Kappa) and **d** spatial distribution of model accuracy (spatial AUC). Black dotted contours represent the 200 m bathymetry (indicative of shelf edge), and canyons are indicated in light grey. There is a complete overlap of validated (red) and thresholded (pink) suitable habitat north of Cape Naturalist (**b**) thus pink polygon is not fully visible

The model output for South West Western Australia indicated high relative habitat suitability centred on the 200 m bathymetry contour covering the outer shelf and slope from Cape Naturaliste to Geraldton and south of Cape Leeuwin (Fig. 4a, b). Suitable foraging habitat was identified as a large semi-continuous area from Windy Harbour (34.8° S) to Geraldton (Fig. 4b) on the slope (79% of suitable habitat) with a smaller amount on the shelf (20%) (Fig. 4b). Spatial Kappa and AUC values indicated the predictive performance of the model was reasonable (≥ 0.4) in most of the areas identified with high habitat suitability (Fig. 4b). Lower values of spatial Kappa and AUC, and thus poor predictive performance (< 0.1), were only observed off the section of the coast between Cape Leeuwin and Cape Naturaliste (Fig. 4c, d).

#### Southern Australia

This subset of the dataset included presence and pseudo-absence data from 11 pygmy blue whales that displayed foraging behaviour in southern Australia (Fig. 1, Figures S2, S3). The gradient boosted model had a global AUC score of 0.95, global Kappa of 0.73 and a correlation between prediction and testing dataset of 0.76, indicating a very good fit. The final model included eight of the variables considered and did not include rugosity (Table 1, Fig. 2c, Supplementary Material 1).

**Fig. 5:**
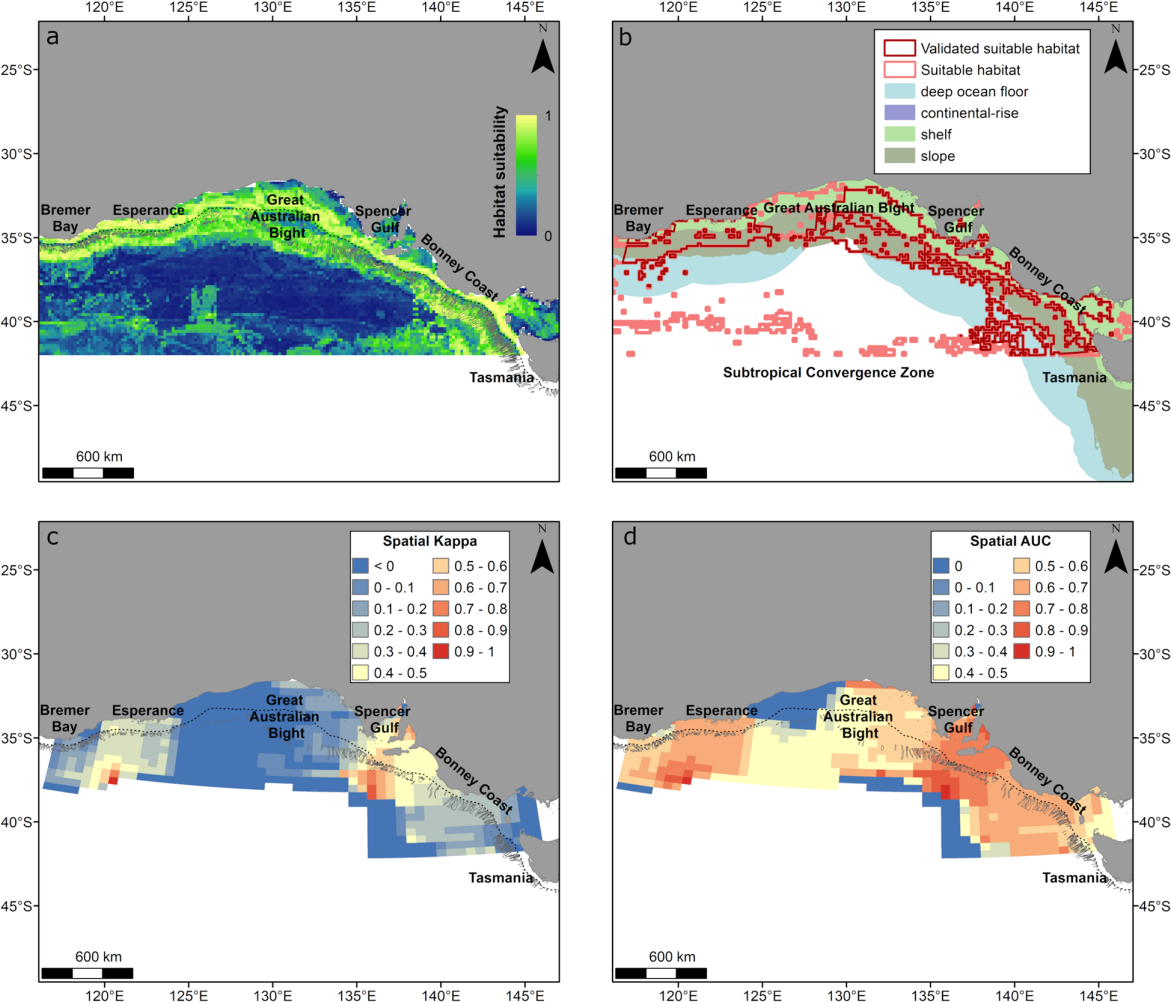
Habitat suitability predicted from the gradient boosted model and spatial accuracy maps for whales displaying foraging behaviour in southern Australia (January–July, November–December). Shown is **a** continuous (0–1) relative habitat suitability, **b** thresholded suitable habitat (pink) and validated suitable habitat (red; suitable habitat restricted to areas with spatial AU and spatial Kappa > 0.4) overlaid with geomorphologic features [32], **c** spatial distribution of model accuracy (spatial Kappa) and **d** spatial distribution of model accuracy (spatial AUC). Black dotted contours represent the 200 m bathymetry (shelf break), and canyons are outlined in light grey. There is a large overlap of validated (red) and thresholded (pink) suitable habitat on the shelf and slope (**b**) thus the pink polygon is not fully visible

For Southern Australia, the model output indicted high relative habitat suitability on the shelf from southwest Western Australia to Tasmania, with highest relative suitability near Bremer Bay, Esperance and in the Bonney Coast (Fig. 5a). Relative habitat suitability was also moderate to high off the shelf, particularly off the Bonney Coast (Fig. 5a). Suitable habitat was represented as a semi-continuous area encompassing both shelf and slope habitats (43% of suitable habitat on the shelf and 48% on the slope), but also by smaller dispersed areas in the deep ocean at the Subtropical Convergence Zone (9% of suitable habitat) (Fig. 5b). Spatial Kappa and AUC values indicated the model had good predictive performance (> 0.4) over the model extent, except for suitable habitat predicted over oceanic areas and parts of the Great Australian Bight (Fig. 5b). Spatial AUC and spatial Kappa values indicated the model performed best off the Bonney Coast and offshore of Bremer Bay, Esperance and in the eastern Great Australian Bight (Fig. 5c, d).

### Foraging/reproduction

#### Southeast Asia region

This subset of the dataset included presence and pseudo-absence data from 11 pygmy blue whales that displayed low move persistence behaviour (breeding, foraging or resting) in the Southeast Asia region (Figure S2). The gradient boosted model had a global AUC score of 0.72, global Kappa of 0.50, and a correlation between prediction and testing dataset of 0.58, indicating a moderate fit. The model included only four of the variables considered for the Foraging/Reproduction model (Table 1, Fig. 2d, Supplementary Material 1).

**Fig. 6:**
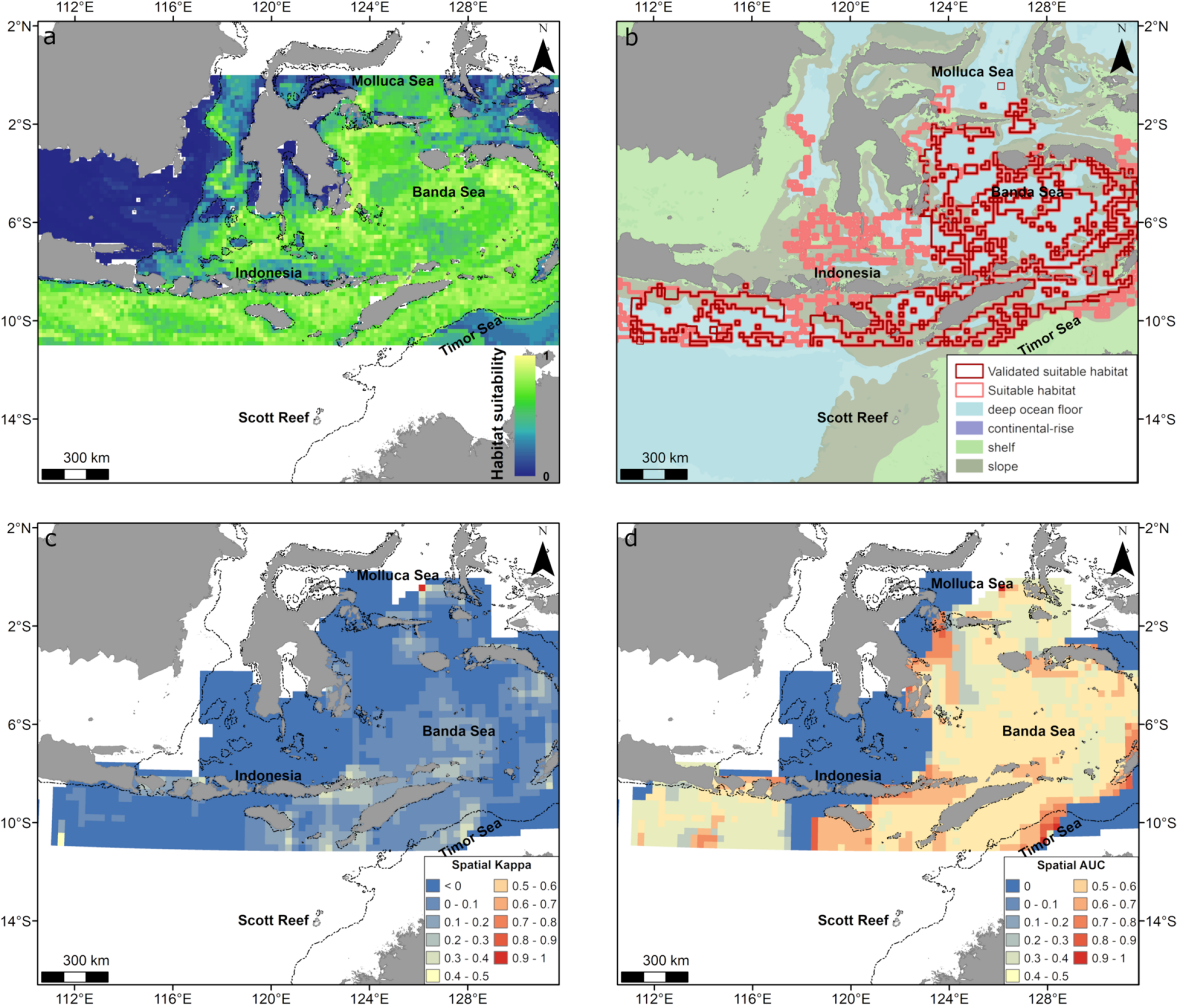
Habitat suitability predicted from the gradient boosted model and spatial accuracy maps for whales in the Southeast Asia region (May–October). Shown is **a** continuous (0–1) relative habitat suitability; **b** thresholded suitable habitat (pink) and validated suitable habitat (red; suitable habitat restricted to areas with spatial AUC and, spatial Kappa > 0.4) overlaid with geomorphologic features [40], **c** spatial distribution of model accuracy (spatial Kappa) and **d** spatial distribution of model accuracy (spatial AUC). Black dotted contours represent the 200 m bathymetry (shelf break). There is a large overlap of validated (red) and thresholded (pink) suitable habitat across the region (**b**) thus the pink polygon is not fully visible

In Southeast Asia, higher relative habitat suitability and suitable habitat occurred in offshore areas across the region that included the Banda, Molluca, Savu and Timor seas (Fig. 6a, b) with 49% of habitat overlapping with slope, 48% with deep ocean and only 2% with continental shelves. The model had some performance issues with low spatial Kappa (< 0.2) across most of the model extent and moderate spatial AUC values (< 0.4). However, spatial Kappa and spatial AUC values were adequate (> 0.4) for areas in the Timor Sea, around Indonesia and in the western Banda Sea (Fig. 6b–d).

### Migration

The subset of the dataset included presence and pseudo-absence from 36 pygmy blue whales that displayed migration movement behaviour (Figure S2, Table S1) including northbound (predominantly) and two southbound tracks (Figure S3). The migration model for Western Australia included five of the variables considered (Table 1, Fig. 2e) and had a global AUC score of 0.87, and global Kappa of 0.46 and a correlation between prediction and testing dataset of 0.57. The migration model for Southern Australia included six of the variables (Table 1, Fig. 2f) and had a global AUC of 0.91, global Kappa of 0.83 and correlation of 0.69, indicating this model performed better than the model for Western Australia.

#### Western Australia

**Fig. 7:**
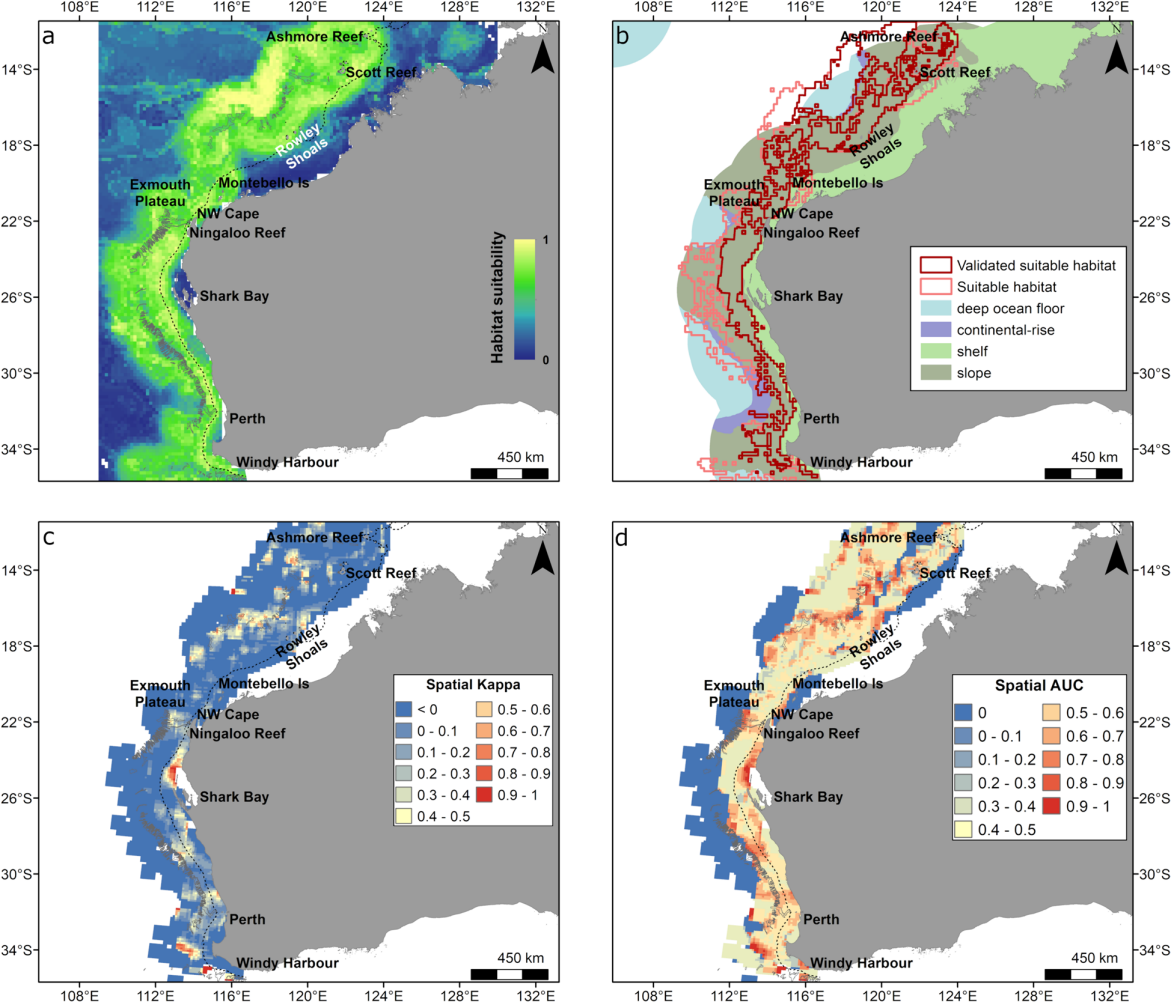
Habitat suitability predicted from the gradient boosted model and spatial accuracy maps for whales displaying migratory behaviour in Western Australia (January to August). Shown is **a** continuous (0–1) relative habitat suitability, **b** thresholded suitable habitat (pink) and validated suitable habitat (red; suitable habitat restricted to areas with spatial AUC and spatial Kappa > 0.4) overlaid with geomorphologic features [32], **c** spatial distribution of model accuracy (spatial Kappa) and **d** spatial distribution of model accuracy (spatial AUC). Black dotted contours represent the 200 m bathymetry (shelf break), and canyons are outlined in light grey. There is a large overlap of validated (red) and thresholded (pink) suitable habitat across the region (**b**) thus the pink polygon is not fully visible

Areas of high relative habitat suitability for migration in western Australia occurred along the shelf break and slope between Windy Harbour and Scott Reef, and also in deep areas offshore of the Rowley Shoals between the latitude of 18° S and 14° S (Fig. 7a, b). In Western Australia, large areas of suitable habitat associated with the slope (76% of suitable habitat) occurred between Cape Leeuwin and Ningaloo Reef (Fig. 7b), with minor predicted habitat (5%) over shelf areas between Perth and Geraldton and adjacent to the Montebello Islands. North of Ningaloo Reef (between 17° S and Ashmore Reef) there was a separation of suitable migration habitat between the deep ocean basin area (19% of suitable habitat) and slope habitat (Fig. 7b). The suitable habitats for migration were mostly supported by the spatial accuracy metrics (Fig. 7c, d) with excellent agreement (spatial Kappa and AUC > 0.8) indicating suitable habitat was validated. However, predicted suitable habitat in offshore areas of western Australia and shelf areas of northwestern Western Australia had lower accuracy and were not validated (Fig. 7b–d).

#### Southern Australia

**Fig. 8:**
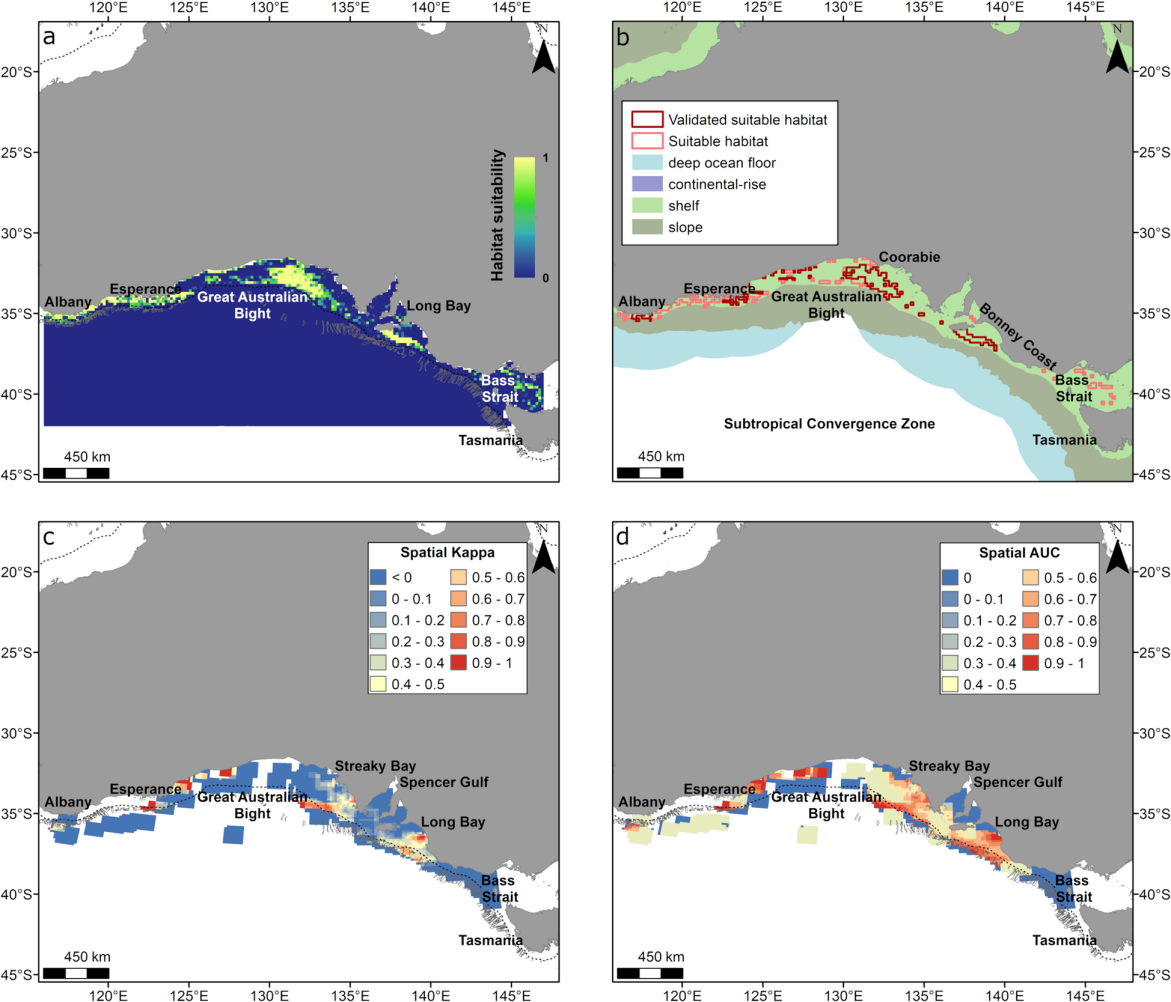
Habitat suitability predicted from the gradient boosted model and spatial accuracy maps for whales displaying migratory behaviour in Southern Australia (January to August). Shown is **a** continuous (0–1) relative habitat suitability, **b** thresholded suitable habitat (pink) and validated suitable habitat (red; suitable habitat restricted to areas with spatial AUC and spatial Kappa > 0.4) overlaid with geomorphologic features [32], **c** spatial distribution of model accuracy (spatial Kappa) and **d** spatial distribution of model accuracy (spatial AUC). Black dotted contours represent the 200 m bathymetry (shelf break), and canyons are outlined in light grey. There is a large overlap of validated (red) and thresholded (pink) suitable habitat across the region (**b**) thus the pink polygon is not fully visible

Areas of high relative habitat suitability for migration in Southern Australia were on the continental shelf from Albany to Long Bay, and western Bass Strait (Fig. 8a, b). Suitable habitat for migration was represented by multiple dispersed areas encompassing mostly shelf habitat (98% of suitable habitat) off the Bonney Coast, the Great Australian Bight and between Albany and Esperance (Fig. 8b). Values of spatial Kappa and AUC indicated good model performance (spatial Kappa and spatial AUC > 0.4) off Albany and Esperance, the Bonney Coast and in coastal areas within the Great Australian Bight near Coorabie (Fig. 8b). However, they indicated that the model performed poorly in the Bass Strait, some areas within the Great Australia Bight and in very shallow coastal areas (Fig. 8c, d).

## Discussion

We provide the first predictions of Eastern Indian Ocean pygmy blue whale relative habitat suitability across most of their known range in relation to foraging and migratory behaviours, with spatially varying accuracy metrics to allow the certainty of the predictions to be assessed. Depth of the water column, i.e., bathymetry, consistently showed high influence on pygmy blue whale relative probability of occurrence for all regions and behaviours. We identified the shelf break and slope as key habitats for pygmy blue whales during foraging and migration in southern and western Australia. Foraging and migrating whales also used shelf habitat in southern and south-western Australia. Once migrating whales reached northern Western Australia and in Southeast Asian waters, they displayed almost exclusive use of slope waters and deep ocean. However, some dynamic variables including sea surface temperature, surface height anomaly and chlorophyll*-a* had a moderate to strong influence on probability of occurrence of whales suggesting suitable habitats may change with different oceanographic conditions and primary production. Importantly, the outputs can be considered to account for some of the limitations associated with defining the extent of area used by a species from satellite tracking data, namely that those outputs infer the area used by the tracked animals only and are thus limited by sample size and tracking duration. While the areas of significance for pygmy blue whales in Australia identified previously [12, 14, 18] are useful for defining the most important areas within their distribution, our outputs here delineate the relative importance of the habitat areas used for foraging, migration and reproduction throughout the majority of the known pygmy blue whale range. The inclusion of robust model validation steps applied here informs where our model performed well and thus provides additional information to advise decision making. For example, they could be considered during the delineation of Biologically Important Areas (e.g., areas where animals display biologically important behaviours that are critical for the survival of the species such as foraging, reproduction, resting) by the Australian Government [31, 67], or be used for environmental impact assessment and defining mitigation of impacts from overlap with threatening activities [17, 68], where high certainty in model prediction is needed.

Overall, our results refine and add to the delineation of suitable habitat for the Eastern Indian Ocean pygmy blue whale [16] (Figs. 3, 4, 5, 6, 7, 8, S11) by increasing sample size, accounting for difference in habitat preference for different behaviours, expanding to include southern Australia and providing information on model accuracy in relation to the spatial predictions presented. Our validated predictions showed preference for foraging habitat along the shelf break (boundary between shelf and slope habitats, indicated by the 200 m bathymetry contour; see Figs. 3 and 4) in Western Australia with expanded use of slope habitat in the North Wwest and some use of shelf in the South West. Whereas, in southern Australia, validated suitable foraging habitat occurred on both the slope and on the shelf with minor use of deep ocean. The preference for shelf break and slope was also observed in Western Australia during migration whereas migration in Southern Australia occurred almost exclusively on the shelf. This finding concurs with previous studies that show that foraging behaviour of pygmy blue whales occurred mostly over the continental shelf in Southern Australia [14] and that foraging pygmy blue whales are often observed very close to the shore in the upwelling system off the Bonney Coast [69]. Our results match a previous analysis of suitable habitat for pygmy blue whales in Western Australia [16] with the analysis showing a strong association to the 1000 m bathymetry contour, which occurs in slope waters along the Western Australia coast. A similar pattern of preference for the shelf break habitat was identified by species distribution models for blue whales along the California coast [10, 33]. Our results for high relative habitat suitability largely overlap with the spatial prediction of a high number of singers in the region during northward migration by [18] using spatial modelling of passive acoustic monitoring data.

Distance to canyons also displayed high relative importance in our models, particularly for foraging in Southern Australia and for migration in the North West, where high probability of occurrence occurred near canyons. This result in the southern Australia model was likely because a high density of canyons occurs along the Bonney Coast supporting localised upwelling, and increased productivity, and thus suitable foraging habitat, within the Great Southern Australian Coastal Upwelling System [70, 71]. The prediction in the North West region, however, showed migration habitat was split between a deep ocean/ outer slope route and a shelf break/inner slope route, falling near but on either side of the canyons. This result is likely influenced by the oceanographic conditions in this area which are driven by the Indonesian Throughflow and the Eastern Gyre [72].

The dynamic variable sea surface temperature was also an important predictor of pygmy blue whale distribution. Although the relationships between probability of occurrence and sea surface temperature were highly variable within and among regions, peaks of probability of occurrence with temperatures of 22–25 °C were observed in most regions in Australian waters, particularly during migration (Supplementary Material 1). A preference for this sea surface temperature range was observed in previous habitat analysis in the region [16] using part of the dataset (from [12]) used in this analysis. This could suggest that there is a potential oceanographic trigger for the start of the northward migration within the foraging areas in Australia, specifically, the strengthening of the Leeuwin Current during March/April which brings warmer waters poleward along the Western Australian coast [73]. This timing matches the start of the northward migration with whales leaving their summer foraging grounds around April [12, 14], and also in June [14], when the Leeuwin current is at its peak [73].

The strong relative influence of the dynamic variables of sea surface temperature and surface height anomaly, and even chlorophyll*-a* (though with overall low relative influence), that can change at fine temporal scales (weeks or days), suggests that suitable habitat will likely change with environmental conditions. This includes not only time of the year or season, but also in relation to oceanographic patterns of upwelling, eddies and during anomalistic events such as heat waves, or cyclones. For example, the presence of foraging pygmy blue whales was associated with increasing chlorophyll*-a* concentration in Timor Leste [30]. Additionally, vocalisations indicative of foraging were strongly related to seasonal wind and coastal upwelling in New Zealand [34] and Chile [74], and blue whale occurrence was associated with thermal fronts off northern Chilean Patagonia [75] and mesoscale oceanographic features in the California Current System [76]. Similarly, foraging, inferred by intensity of vocalisations, seemed to decrease during a heatwave [50]. The influence of dynamic variables calls for some caution in the inferences from static maps of suitable habitat or biologically important areas often used for species management. Although we included month of the year in our models, the limited sample size (number of tracks and location points) for some months (most of the data restricted to April to June; see Fig. 1b) prevented us from providing monthly maps of suitable habitats. To overcome this issue and include a temporal aspect to our results, our predictions used the average of the dynamic variables across the full temporal extent of the dataset within each modelled region and subset of the data. This was possible because pygmy blue whale showed clear seasonal movements within and between regions (see Fig. 1). Thus, the maps of suitable habitat we provide can be considered as the average suitable habitat for pygmy blue whales during the time they are present in each section of their distribution range in the Eastern Indian Ocean.

Our approach of splitting the data into regions for modelling also accounts for some of the temporal/seasonal components of pygmy blue whale habitat selection [77] to avoid extreme extrapolation that can decrease model performance. In the future, with a larger sample size, we hope to be able to make our model predictions per month and ideally they would be predicted dynamically [10]. Such dynamic predictions can allow for more efficient management of interactions between migratory species and potential threatening human activities than static areas. The concept of dynamic ocean management was developed to account for the shifting and complex nature of the ocean that can integrate animal tracking, remote sensing and advanced modelling techniques, with the potential of making predictions in near-real time [78, 79]. Information obtained from animal tracking is well suited for such a management approach, particularly for threatened and/or highly mobile species [10, 80], when large sample sizes are obtainable. For example, dynamic ocean models have been successfully developed for the California Current Ecosystem as a fisheries management tool to minimise bycatch of threatened and protected species by the drift gillnet fishery [81, 82].

Global AUC and Kappa values for the models were good overall, however, spatial Kappa and AUC showed that the accuracy of predictions varied in space. There were several areas where high habitat suitability was predicted but for which the spatial accuracy metrics were low, namely foraging at Scott and Ashmore reefs, the offshore waters between Capes Naturaliste and Leeuwin, parts of the Great Australia Bight and oceanic waters in Southern Australia, and also migration habitat in southern Western Australia (between Esperance and Albany) and Bass Strait. Thus, there is low confidence in the prediction of suitable habitat in these areas. This result occurred because we had no/limited presence points for those behaviours/areas. Having a greater sample size of tracked individuals would assist in determining the importance of these areas as our sample size is still small and thus likely insufficient to characterise habitat preferences for the entire stock or population [83, 84]. As such, it may also be more influenced by individual preferences [83] not accounted for in our models. Although other modelling methods can account for individual (ID) variability as a random effect (mixed models), our preliminary analysis showed that boosted trees performed better than generalised additive models and that the addition of a predictor of whale ID (proxy for assessing the potential random effect of individual whale preferences) did not significantly influence the results of our models (Supplementary Material 1). Our results are also likely influenced by some of the biases inherent to tracking data such as differences in deployment duration, resulting from premature tag detachment, or failure [85]. Other factors at play are that we had low temporal coverage in some of the modelled regions and low covariate resolution (1, 4 and 17 km—see Table 1), particularly freely available remote-sensed data for offshore waters (https://portal.aodn.org.au/), and missing values of covariate data which can have a large impact on the accuracy of model predictions [86]. The issue of resolution is a main constraint of modelling species distributions with remote-sensed data, typically available at relatively large grid sizes, compared to the scale of use by the animals [86, 87]. This issue is not easily resolved for wide-ranging and highly mobile marine species as we rely on remote-sensed data to model the relationship between species occurrence and environment over the extensive areas they use. To achieve this goal for pygmy blue whales in Australia, we must continue to increase sample size of tracked whales, including the southward migration [12, 18] and incorporate other sources of data such as localised structured surveys (for areas where pygmy blue whales are found closer to shore such as the Bonney Upwelling in Southern Australia [[14, 69]; Fig. 5], South West Western Australia [[13, 88]; Fig. 4] and Ningaloo Reef [Fig. 3]), and improve the resolution of the environmental variables used in our modelling; for example, by complementing remote-sensed data with in situ measures [89]. Although it is impossible to obtain in situ measures for the entire distribution, improved environmental data within the important areas defined previously [18] or in areas of high exposure to threats [17], could assist in the development of high-resolution dynamic models. In the interim, our outputs could inform recovery planning for this species such as the existing pygmy blue whale distribution maps and biologically important areas (see [31, 67]) that are currently under review. We show that large extents of suitable habitat are available for pygmy blue whales that can be used to inform the delineation of possible foraging habitat, with results from analysis of tracking data by [18] and [14] informing high-use areas. Most importantly, our maps of model accuracy indicate areas where data is still limited, hence where interpretation of habitat maps and telemetry data should be made with caution. In these areas, our predictions can be combined with other data sources such as passive acoustic monitoring, direct observations, and structured survey data.

## Conclusions

The averaged spatial predictions and validated relative suitable habitat maps produced here can be useful for informing current management actions in Australia determined by the Conservation Management Plan for the Blue Whale [31], such as the need to delineate spatial distribution of foraging and migration habitat for the species. However, the importance of dynamic covariates in the model indicates that the relationship between whale occurrence and environment is not static in time or space, and so the habitat suitability maps presented here must be considered within the temporal context of each dataset.

In summary, this is the first quantitiative delineation of suitable habitat across the known distributional range of pygmy blue whales in the Eastern Indian Ocean provided with spatially varying metrics of model accuracy. Thus, our outputs provide an assessment of habitat suitability in relation to foraging and migratory movements as well as an assessment of how reliable our prediction maps are across the pygmy blue whale range. This information is needed by State and Commonwealth regulators, and the offshore industries, to support effective impact assessment to evaluate the potential impacts of human activities on the pygmy blue whale population in the Eastern Indian Ocean.

### Supplementary Information

Supplementary Material 1.

## Data Availability

Raw tracking data collected by the Australian Institute of Marien Science can be accessed in Zoatrack (https://zoatrack.org/) and the AIMS Data Centre (https://apps.aims.gov.au/metadata/search). Tracking data collected by the Australian Antarctic Division can be accessed through their Data Centre (https://data.aad.gov.au/).
