# Supplementary material for: Predicting suitable habitats for foraging and migration in Eastern Indian Ocean pygmy blue whales from satellite tracking data
Source: Mov Ecol. 2024 Jun 7;12:42. doi: 10.1186/s40462-024-00481-x (PMC11157879; doi:10.1186/s40462-024-00481-x)
Supplement: Supplementary file 1 — Supplementary Material 1. [file 40462_2024_481_MOESM1_ESM.pdf]

Method

Model comparison

We attempted to build generalised additive mixed models that included a random effect to compare their predictive power against boosted random trees. The GAMM framework can account for the inherent sample bias that arises due to repeated measures (locations) from the same tagged individuals and autocorrelation associated with tracking data (sequential data in time and space) by including tagged individual (ID) as a random effect within the model. In this framework we used a binomial error distribution with presence and pseudo-absence as the response variables, as we did for the gradient boosted models. The models were done for each migration and resident (foraging and breeding) location data separately. Model outputs were evaluated by the area under the receiver operating curve (AUC; (1)) to assess the ability of the model to discriminate between presence and pseudo-absence points. The AUC values range between 0 and 1, with a value of 0 indicating a complete mismatch between the model prediction and actual presence data, and a value of 1 indicating a complete alignment between the prediction and the presence data.

We also tested the influence of individual variation in habitat selection by comparing gradient boosted models with whale ID as predictor and without it.

Results

Generalised linear models

The top-ranked models and associated parameters for each behaviour/region are listed in Table S1, showing the most important (Table S3). Percent deviance explained was low (<15%) and AUC scores were considerably lower than those estimated for gradient boosted models (Table S3).

Effect of adding a random effect of whale ID

Gradient boosted model containing ID as a random predictor variable had similar global AUC and Kappa estimates than the model without the variable (Table S4). There was also no change in the overall patterns of partial plots for the top variables between model configurations (Figure S12 vs Figure S5-S10), thus we selected to use the simpler models, without ID, in our analysis.

Foraging

North West Western Australia

Depth of the water column (*bathy*) was the most important variable (Figure 2a), followed by Surface height anomaly (*sla*), Sea surface temperature (*sst*) and Distance to canyon (*dist_can*) (Figure 2a). Month had the lowest influence in the model (Figure 2a). Partial plots indicated that probability of occurrence increased with increasing Depth of water column (*bathymetry*) until of about 930 m depth, and then decreased with increasing depth (Figure A4). Probability of occurrence was also greatest with Surface height anomaly of 0.07m, Sea surface temperature between 19° and 26°C, with a secondary peak at 30°C, but also higher log Chlorophyll-a concentration and increasing Rugosity (Figure A5). Higher probability of occurrence was associated with areas adjacent to canyons but also at distances of 80 km and 130 km, Standard deviation of sea surface temperature above 1.5°C, and variable Standard deviation of surface height anomaly, with peaks at < -0.1, 0, 0.18 and 0.25 m. Higher probability of occurrence (displaying foraging behaviour) was also associated with the months of June and July (Figure A5, Figure 1b).

South West Western Australia

The top variable was Depth of the water column and had a relatively larger influence than the least important variables (29.5% and 2.2%, respectively) (Figure 2b). The next most important variable in relation to its relative influence in the model, was Sea surface temperature (Figure 2b). After these main variables, multiple variables had similar relative influence including Surface height anomaly and its standard deviation, the Standard deviation of the sea surface temperature, Log Chlorophyll-a, Rugosity and Distance to canyon, with Month having the lowest relative influence (Figure 2b). Probability of occurrence increased with increasing Depth of the water column peaking at 115 m depth (and declining after that), and Surface height anomaly of 0.08 m and its standard deviation of 0.05 and 0.2 m (Figure A6). The relationship with Sea surface temperature was variable with the main peaks at 13°, 16.4° and 25.3° (Figure A6). Higher probability of occurrence was also associated

with Standard deviation of sea surface temperature of 1.3°C, increasing Rugosity (> 400), and distance of 12 km to canyons (Figure A6). The relationship between whale occurrence and Log Chlorophyll-a was more complex with probability of occurrence first decreasing and then increasing with increasing chlorophyll-a concentration (Figure A6).

Southern Australia

Depth of the water column had the highest relative influence, followed by Distance from canyons and Sea surface temperature (Figure 2c). The variable with the lowest influence was Standard deviation of the sea surface temperature (Figure 2c). Probability of occurrence was highest in areas with water depths around 100 m with occurrence dropping off to moderate levels in deeper waters and declining in water deeper than 4600 m (Figure A7). High probability of occurrence was also predicted close to canyons (peaks at 0 and 50 km) and with variable Sea surface temperature with peaks at 11°, 14° and 23°C (Figure A7). Higher probability of occurrence was also associated with increasing Surface height anomaly, although multiple peaks at -0.05, 0 and 0.012 m were also observed (Figure A7). Higher probability of occurrence was associated with extreme values of the Standard deviation of surface height anomaly (< - 0.1 and > 0.3 m) and increasing standard deviation sea surface temperate and (log) chlorophyll-a concentration (Figure A7). Highest probability of occurrence occurred between June and August (Figure A7).

Foraging/reproduction

Southeast Asia

Surface height anomaly had the highest relative influence, closely followed by Sea surface temperature, Depth of the water column (*bathymetry*) and then Month (Figure 2d). The relationship between probability of occurrence and environment was extremely variable but there was a general increase in probability of occurrence with increasing and positive Surface height anomaly values and highest probability of occurrence with Sea surface temperatures around 15°C but also 29°C (Figure A8). The highest probability of occurrence coincided with deep water depths (~700 to 7000 m) and

declined in waters shallower than 550 m (Figure A8) and was highest during June, September and October (Figure A8).

Migration

western Australia

For the Western Australia migration model, Distance to canyons was the top variable, but was closely followed by the Standard deviation of surface height anomaly, Surface heigh anomaly, Depth of the water column and with Sea surface temperature as the lowest influence (Figure 2e, Supplementary material). In Western Australia, probability of occurrence during migration was highest between 26 km and 78 km from canyons, Surface height anomaly below -0. 2 m and between -0.1 m and 0.2 m, and its standard deviations between -0.01 and 0.07 m (Figure A9). Probability of occurrence had a variable relationship with Depth of the water column and sea surface temperature with peaks at depths at 6000 m and 250 m, and Sea surface temperatures between 29° and 17°C (Figure A9).

Southern Australia

For the Southern Australia migration model, the highest ranked variable, Depth of the water column had larger relative influence compared to other variables (39%), with Surface height anomaly and Distance to canyons making up the next top two most important variables, and Standard deviation of the surface height anomaly with the lowest influence (Figure 2f, Supplementary material).

In Southern Australia, probability of occurrence of whales displaying migratory movement behaviour increased with decreasing Depth of the water column up to 230 m deep, with probability of occurrence decreasing after that (Figure A10). Probability of occurrence also increased with Distance to canyons with peaks at 46 km and 200 km from canyons, and Surface height anomaly of -0.02 m and 0.02 m (Figure A10). Probability of occurrence was also highest in area with Rugosity between

300-500, increasing Standard deviation of surface height anomaly, and Sea surface temperatures between 22 and 25°C (Figure A10).

Supplementary Tables

Table S1 Summary of the datasets compiled for the analyses including deployment location, number of tags deployed (n), deployment year, tag type, median (and range) deployment length in days, and the type of location data provided by the tags.

Deployment location	n	Year	Tag type	Deployment length (days)	Location data type
Perth Canyon, WA	9	2009, 2011	SPOT	32 (6 - 141)	Argos
Perth Canyon, WA	10	2021,2022	LIMPET	19 (8-80)	Argos and GPS
Bonney Upwelling, SA	13	2015	SPOT/SPLASH	60 (8 - 384)	Argos
Ningaloo, WA	6	2019, 2020	LIMPET	52 (14 -122)	Argos and GPS

Table S2 Summary of the datasets for each modelled region including the sample size of tagged whales represented in each region (N), temporal extent tracking of the data included in the analysis (month – year combinations) and assigned behaviour (see Methods section for details).

Region	N	Months	Year	Behaviour
North West	17	April<br>May<br>June<br>July<br>August<br>September	2011,2021<br>2009,2015,2020-2022<br>2015,2019,2020,2022<br>2019<br>2022,2022<br>2020	Foraging
South West	20	March<br>April<br>May<br>June<br>November	2011<br>2009,2011,2021,2022<br>2015, 2021, 2022<br>2015<br>2015	Foraging
Southern Australia	11	January-December	2015-2016	Foraging
Southeast Asia region	11	May<br>June<br>July<br>August<br>September<br>October	2011<br>2011, 2015,2020,2022<br>2011,2020-2022<br>2009,2015,2020,2022<br>2015,2020<br>2015	Foraging/Reproduction
Western Australia	27	March<br>April<br>May<br>June<br>July<br>August<br>September-October<br>November	2011<br>2009,2011,2021,2022<br>2009,2011,2015,2020-2022<br>2009,2015,20-2022<br>2009,2019,2020-2022<br>2009,2015,2020<br>2015-2020<br>2015	Migration
Southern Australia	11	January-March<br>April-July<br>November-December	2015-2016<br>2015<br>2015	Migration

Table S3. Top-ranked generalised additive mixed-effects model (GAMM), from the suite of models fitted to examine the relationship between pygmy blue whale presence and environmental variables showing percent deviance explained (DE%) and area under the receiver operating curve (AUC). We also show AUC for gradient boosted models (GBM) for comparison.

Model formula*	Region	Behaviour	DE%	AUC	AUC (gbm)
**P/A ~bathy+dist+height**	North-west	Foraging	10%	0.77	0.85
**P/A ~bathy+sst+rug**	South-west	Foraging	12%	0.66	0.86
**P/A ~bathy**	Southern Australia	Foraging	11%	0.69	0.95
**P/A ~bathy+height+rug**	Southeast Asia	Breeding	5%	0.57	0.72
**P/A ~bathy+month**	Australia	Migration	12%	0.43	0.87/0.91

*P = presence (actual track), A = pseudo-absences (simulated tracks), Bathy= bathymetry, dist = distance from canyon, height = sea surface height anomaly, sst = sea surface temperature, rug = rugosity

Table S4. Comparison of ID

		Gradient boosted model without ID as predictor		Gradient boosted model with ID as predictor	
Region	Behaviour	Global AUC	Global Kappa	Global AUC	Global Kappa
North West	Foraging	0.85	0.4	0.85	0.4
South West	Foraging	0.86	0.5	0.76	0.6
Southern Australia	Foraging	0.95	0.7	0.91	0.6
Southeast Asia	Foraging	0.72	0.5	0.94	0.6
Southern Australia	Migration	0.91	0.7	0.98	0.8
Western Australia	Migration	0.87	0.6	0.90	0.5

Supplementary Figures

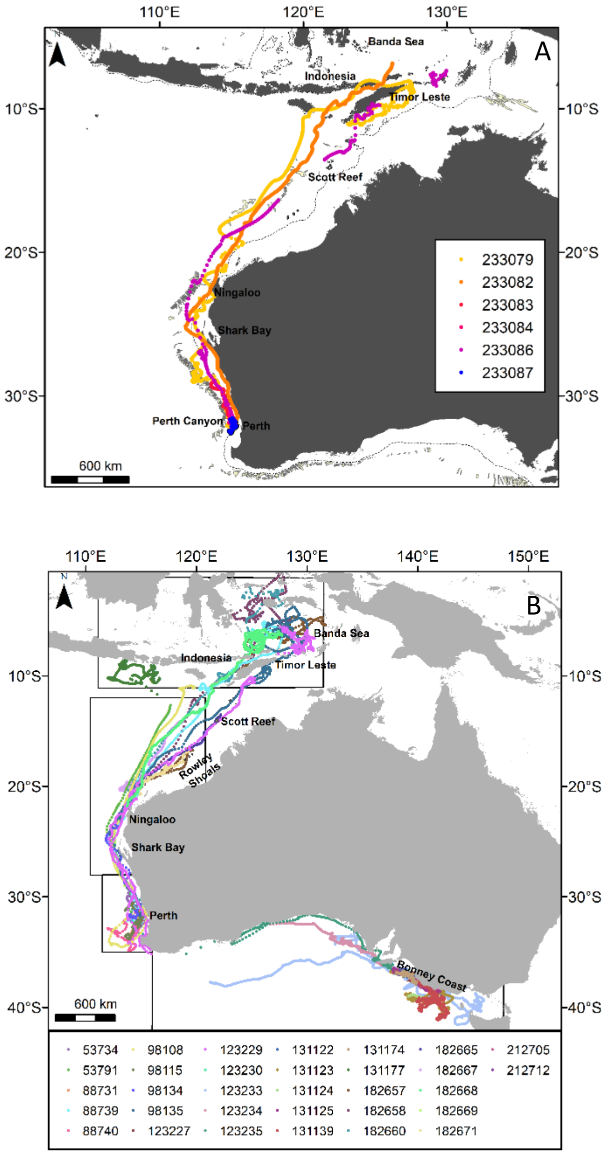

Figure S1. Satellite tracks of six pygmy blue whales (ID numbers shown in legend) new tag deployment in 2022 in the Perth Canyon (A) and from existing tag deployments (B). Grey polygons in (A) are canyons and dotted line is the 200 m bathymetric contours and black polygons in (B) are the four regions for which habitat modelling was undertaken (see Figure 1).

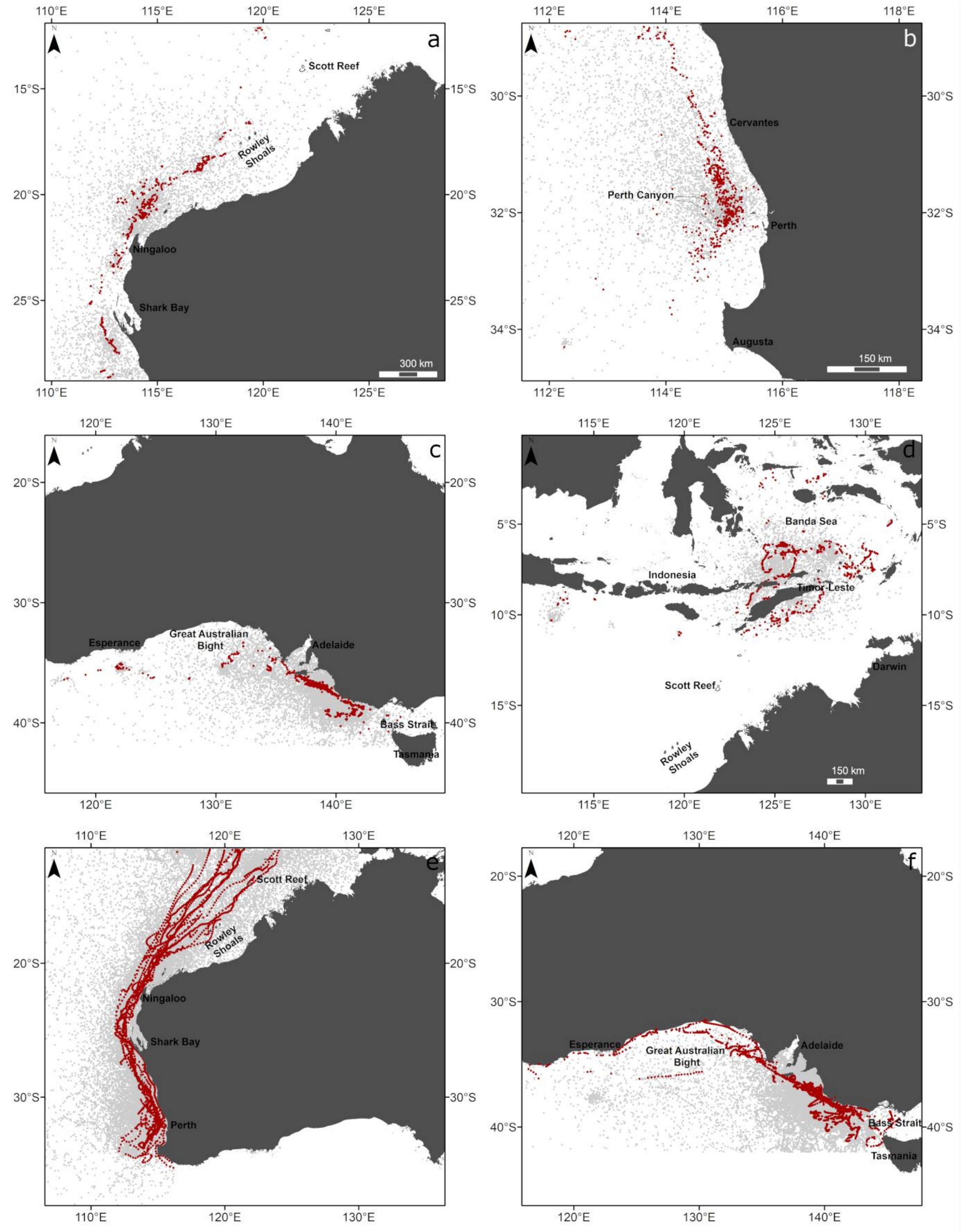

Figure S2. Maps of pygmy blue presences (satellite tracking data) in red and simulated tracking data shown in grey points (pseudo-absences) for foraging areas in North West WA (a), South West WA (b), Southern Australia (c), foraging/ reproduction area in the Southeast Asia region (d), and migration off Western Australia (e) and Southern Australia (f). The presence and pseudo-absence points were the input data for the gradient boosted models to predict probability of occurrence and habitat suitability in each of the study regions.

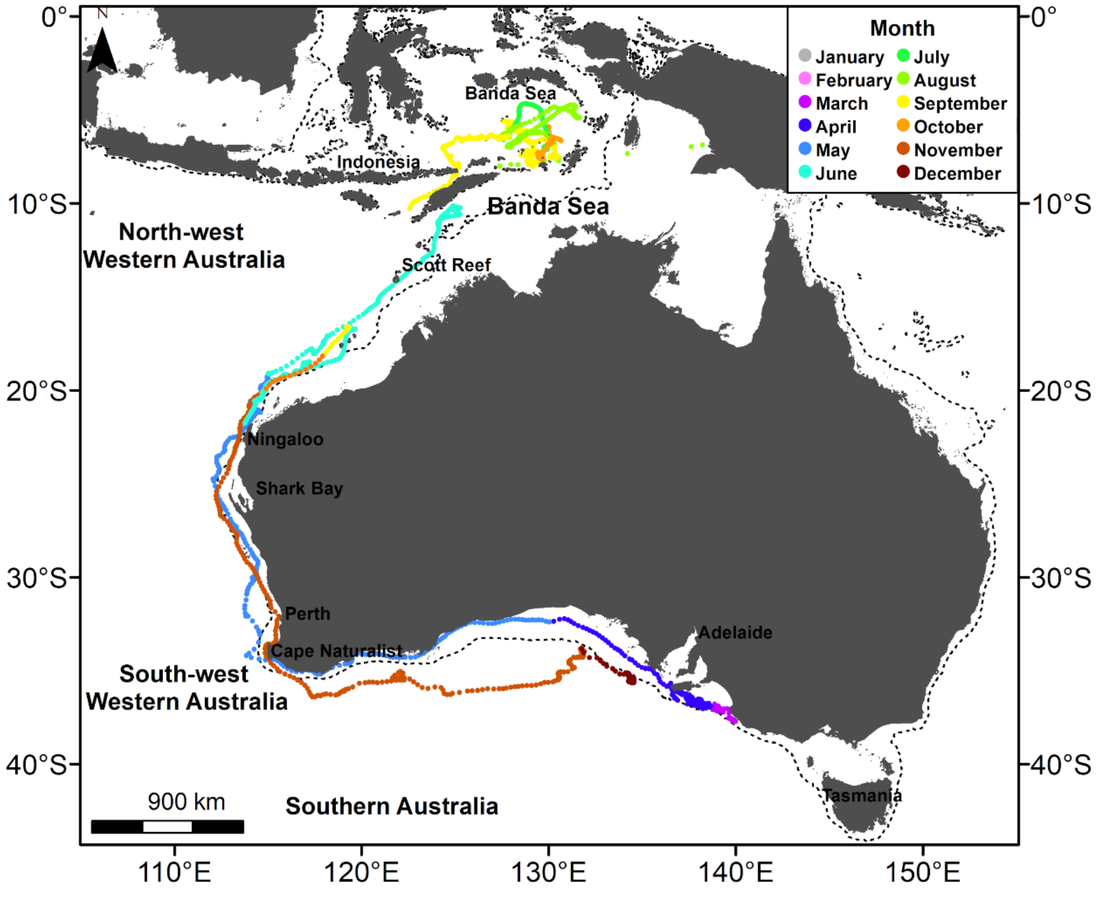

Figure S3 State-space modelled satellite tracks of two pygmy blue whales that provided data on the southern migration (123229 tagged in Southern Australia and 182657 tagged at Ningaloo). Tracks colour coded by month of the year. Dotted black contour represents the 200 m bathymetry contour as a proxy of the shelf edge in Australia.

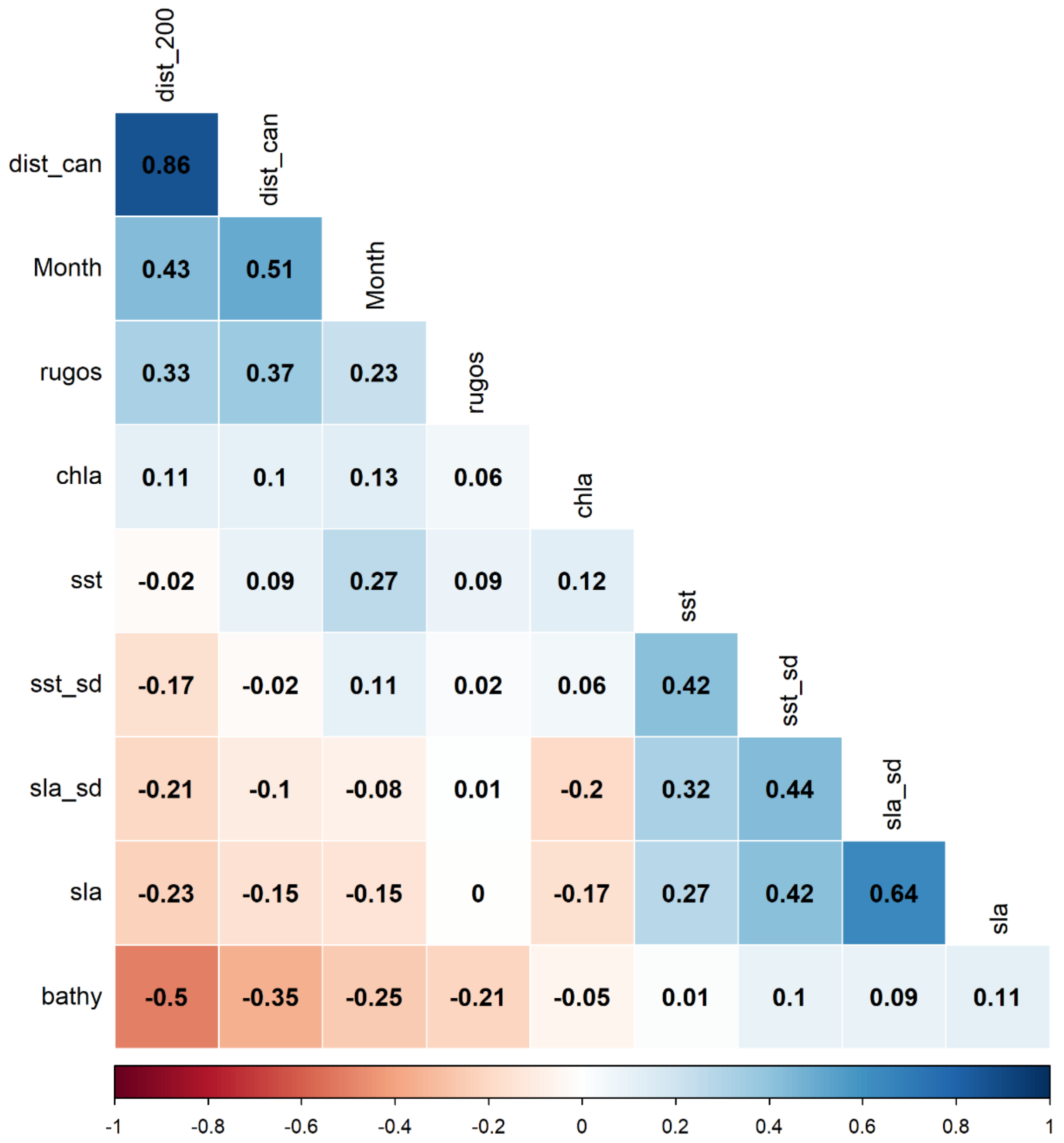

Figure S4 Collinearity matrix of all predictor/environmental variables (Table 2) used during model selection for the modelling of pygmy blue whale habitat suitability for each region and behaviour. Each row and column represent a predictor and the cell of each row-column intersection indicates how correlated these are, with darker colours indicating high correlation, blue indicating a positive relationship and red indicating a negative relationship and correlation coefficient printed for each combination. *Bathy* = Depth of water column (Bathymetry); *sla* = Sea surface height anomaly; *sla_sd* = Standard deviation of the sea surface height anomaly; *sst_sd* = Standard deviation of the sea surface temperature; *sst* = Sea surface temperature; *chla* = Log Chlorophyll-a; *rugos* = Rugosity; *dist_can* = Distance from canyons; and *dist_200* = Distance from the 200 m bathymetry contour.

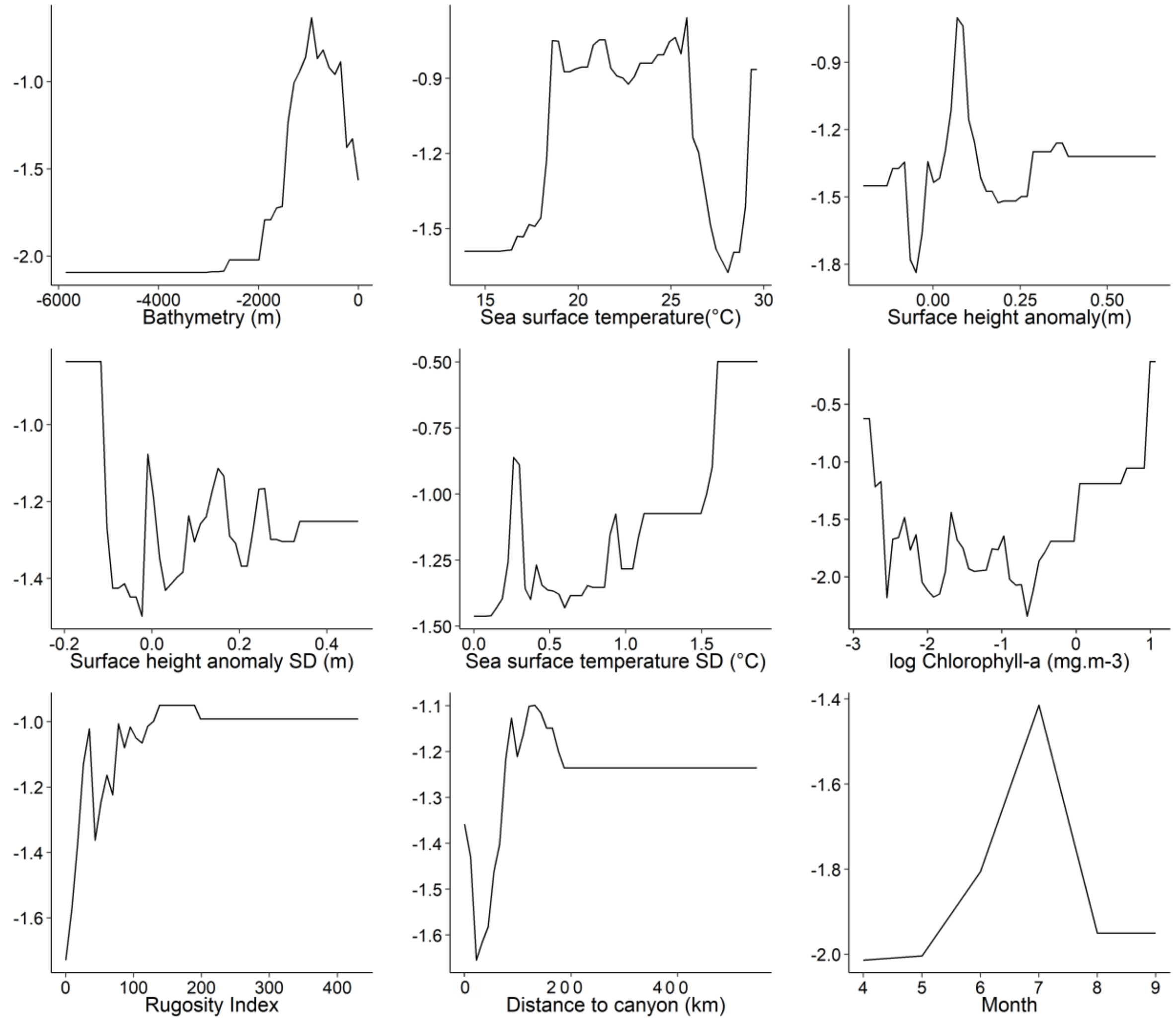

Figure S5 Partial plots showing the relationship between probability of occurrence and the environmental variables included in the final gradient boosted model (Figure 2) for foraging in North-West Western Australia.

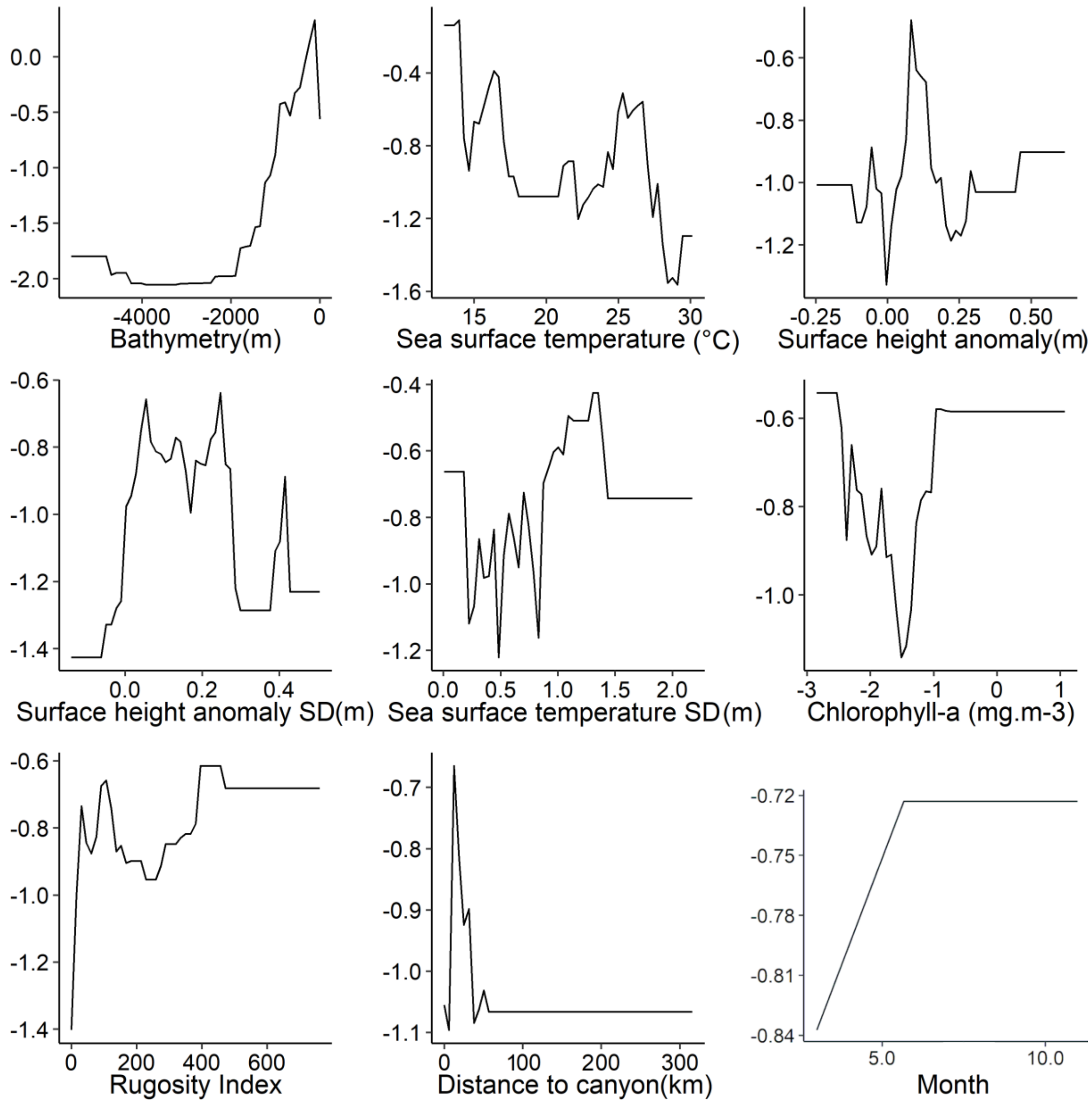

Figure S6 Partial plots showing the relationship between probability of occurrence and the environmental variables included in the final gradient boosted model (Figure 2) for foraging in South West Western Australia.

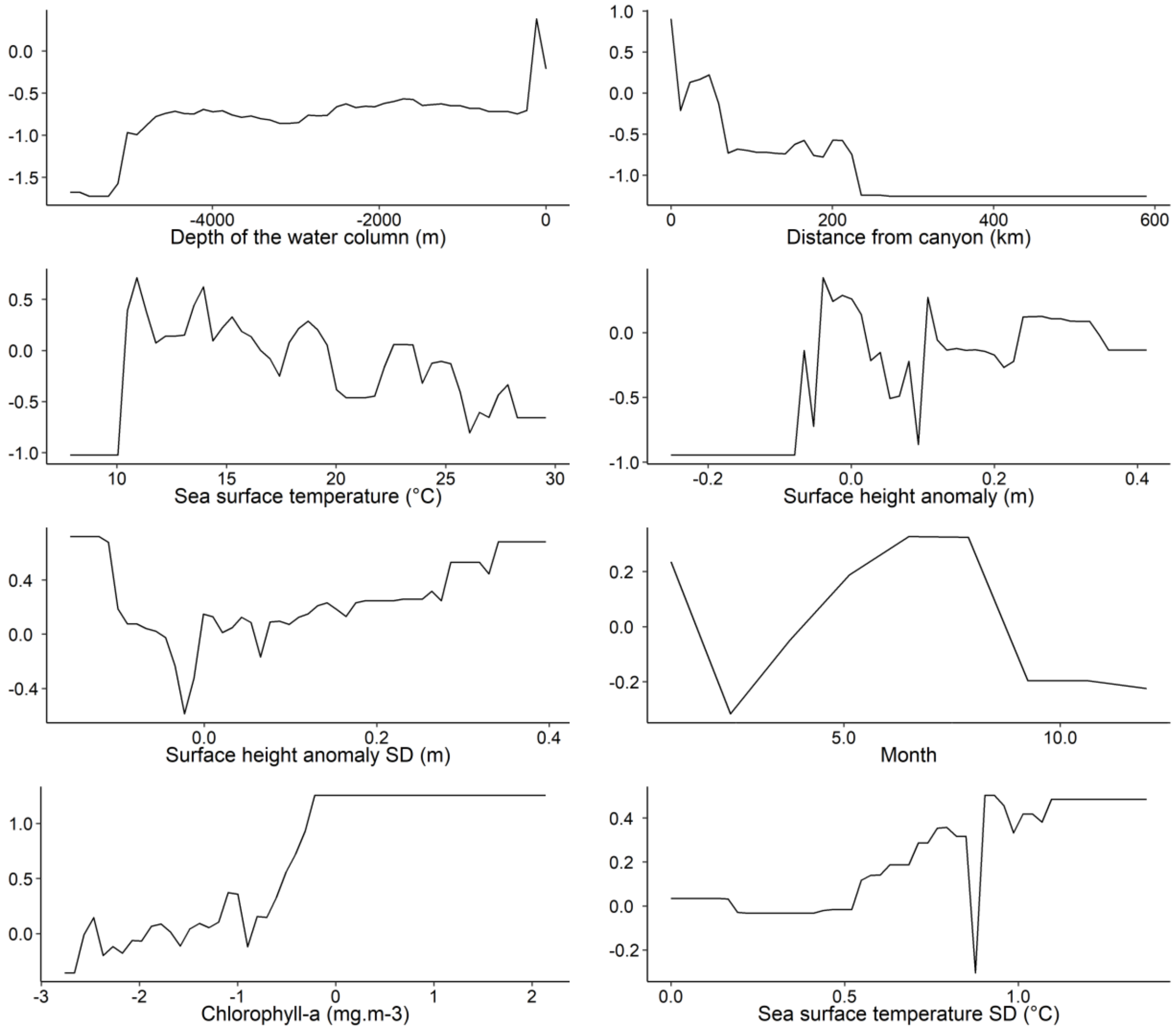

Figure S7 Partial plots showing the relationship between probability of occurrence and the environmental variables included in the final gradient boosted model (Figure 2) for foraging in Southern Australia.

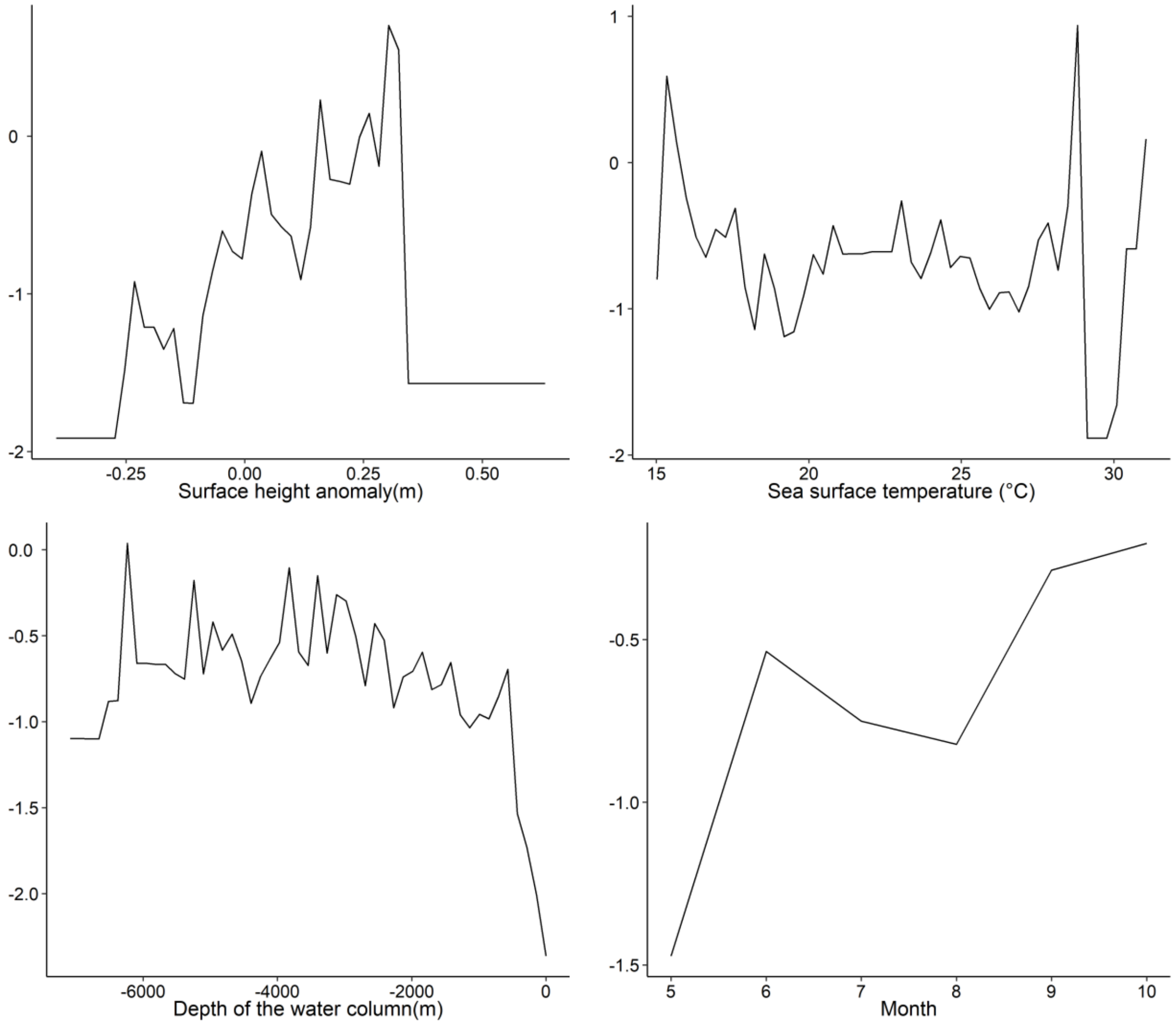

Figure S8 Partial plots showing the relationship between probability of occurrence and the environmental variables included in the final gradient boosted model (Figure 2) for foraging/reproduction in Southeast Asia.

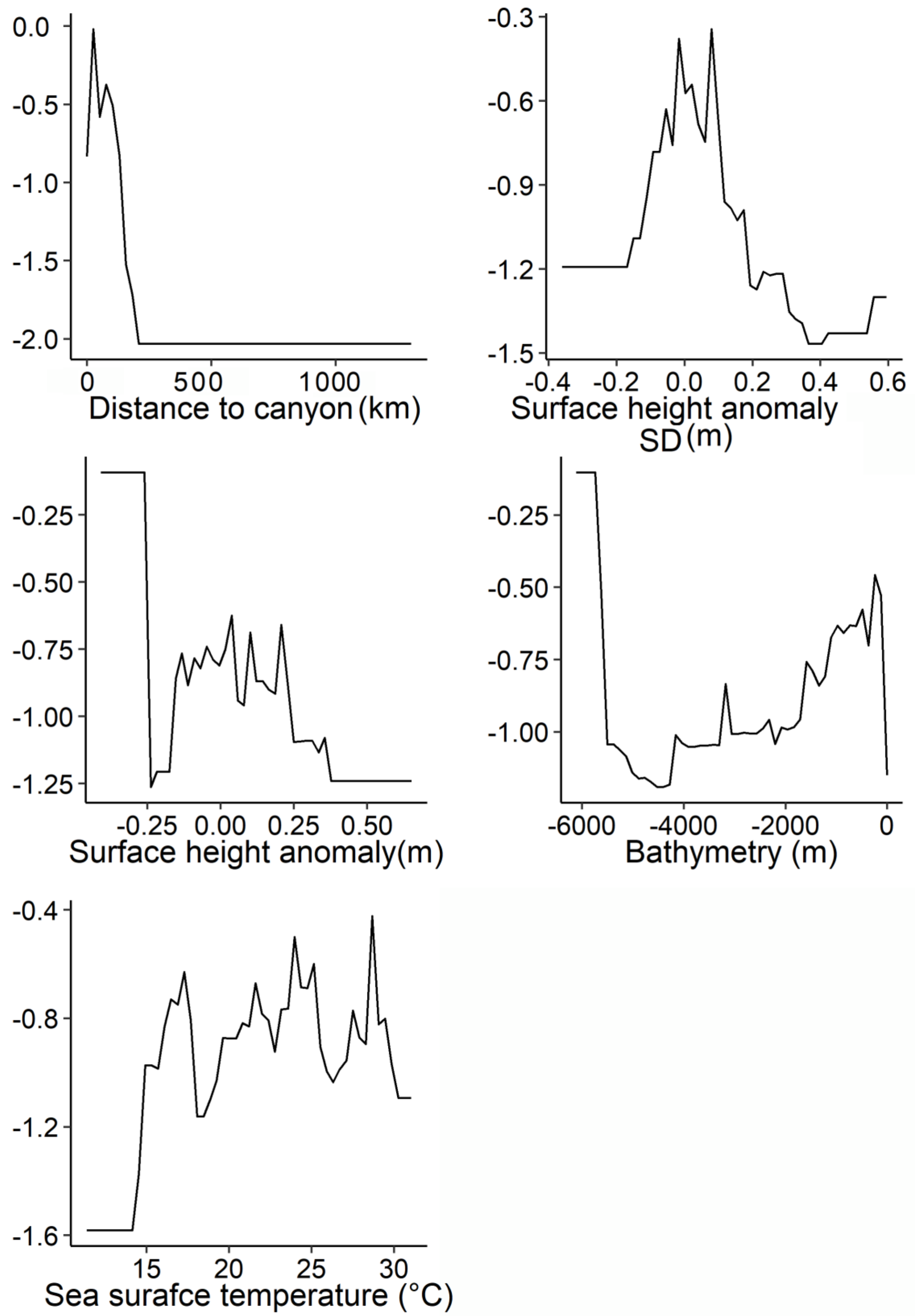

Figure S9 Partial plots showing the relationship between probability of occurrence and the environmental variables included in the final gradient boosted model (Figure 2) for migration in western Australian.

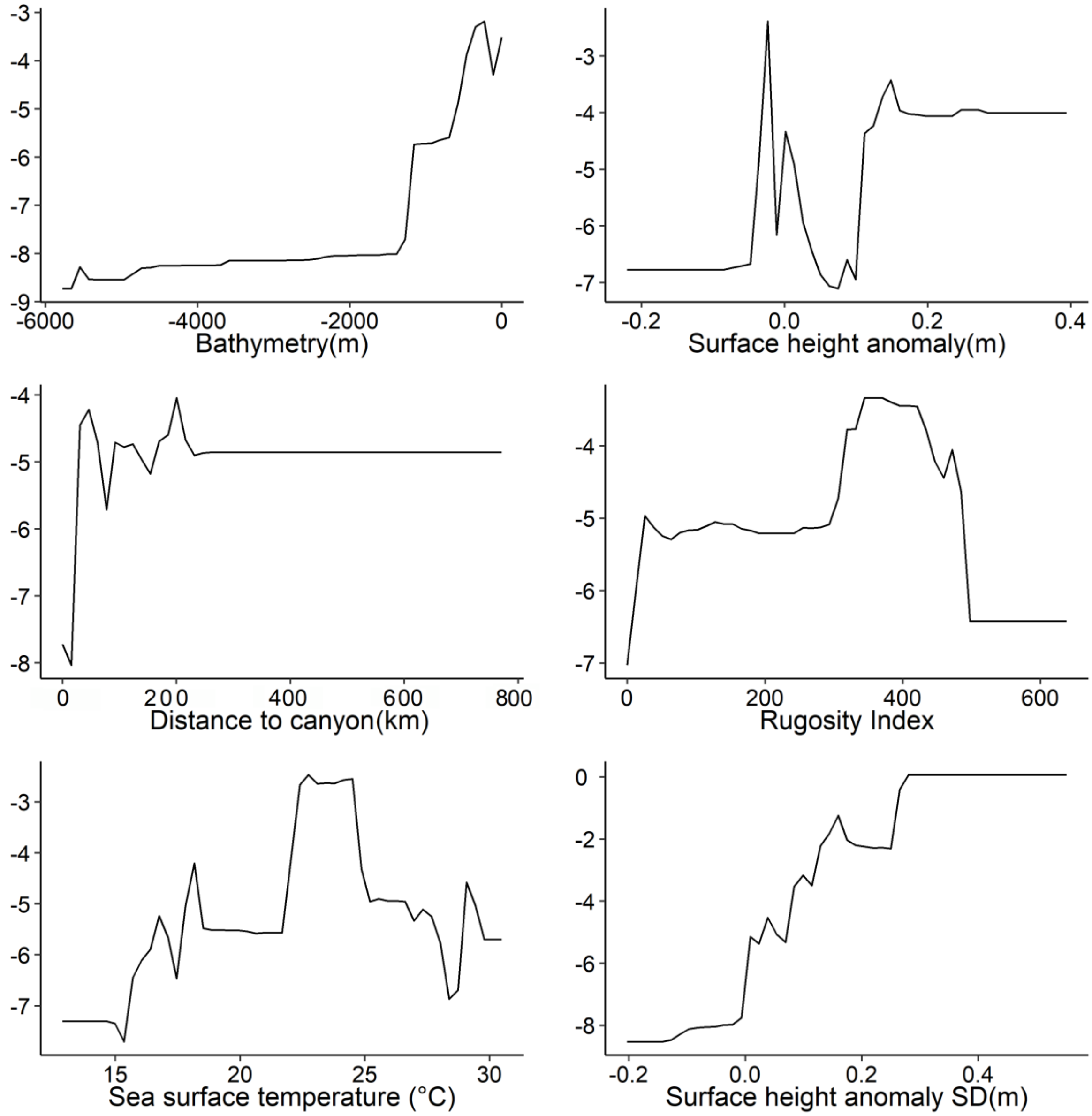

Figure S10 Partial plots showing the relationship between probability of occurrence and the environmental variables included in the final gradient boosted model (Figure 2) for migration in southern Australia.

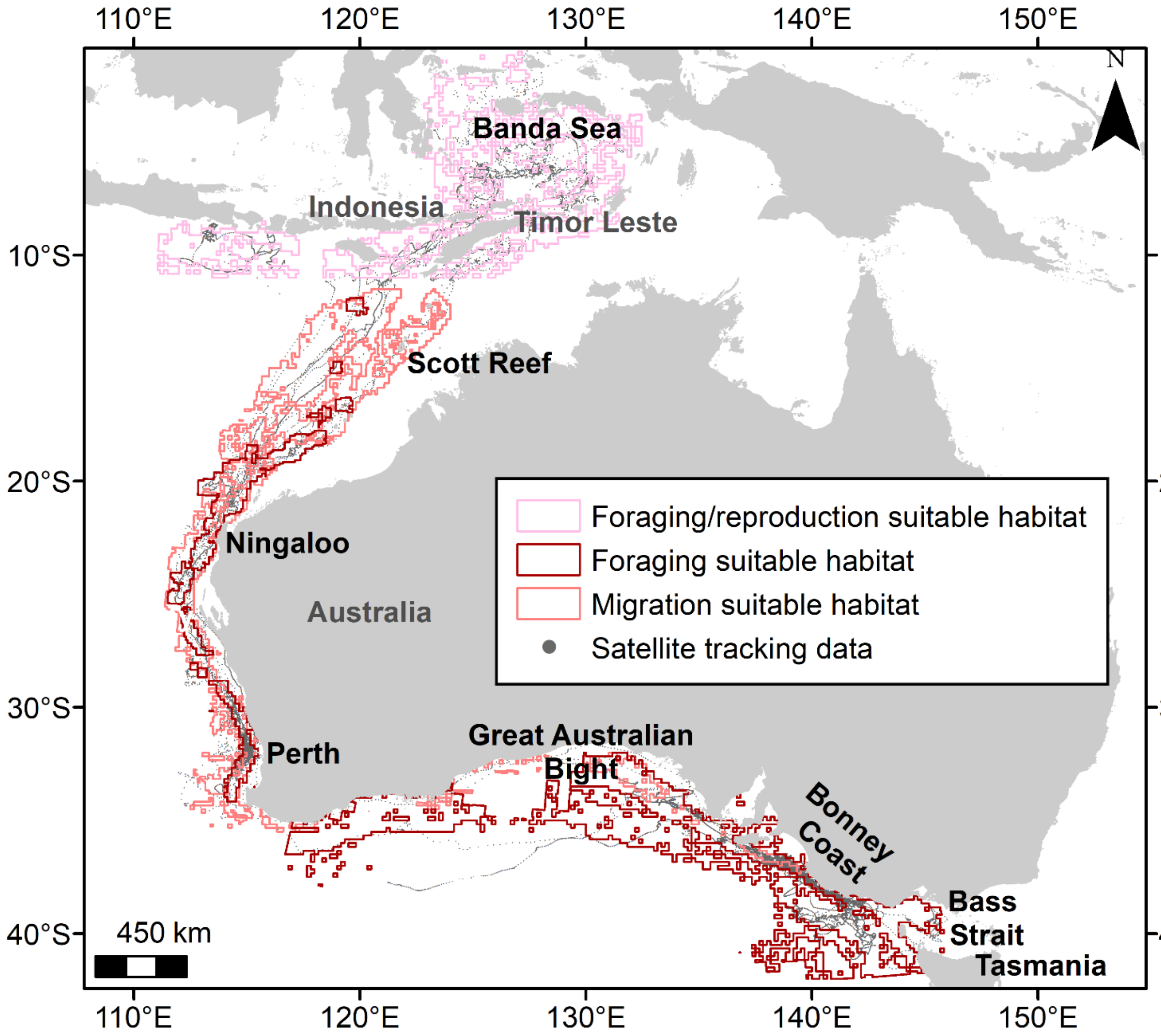

Figure S11 Validated suitable habitat for foraging/reproduction (pink), foraging (dark red) and migration (light red), and satellite tracking data for Eastern Indian Ocean pygmy blue whales.

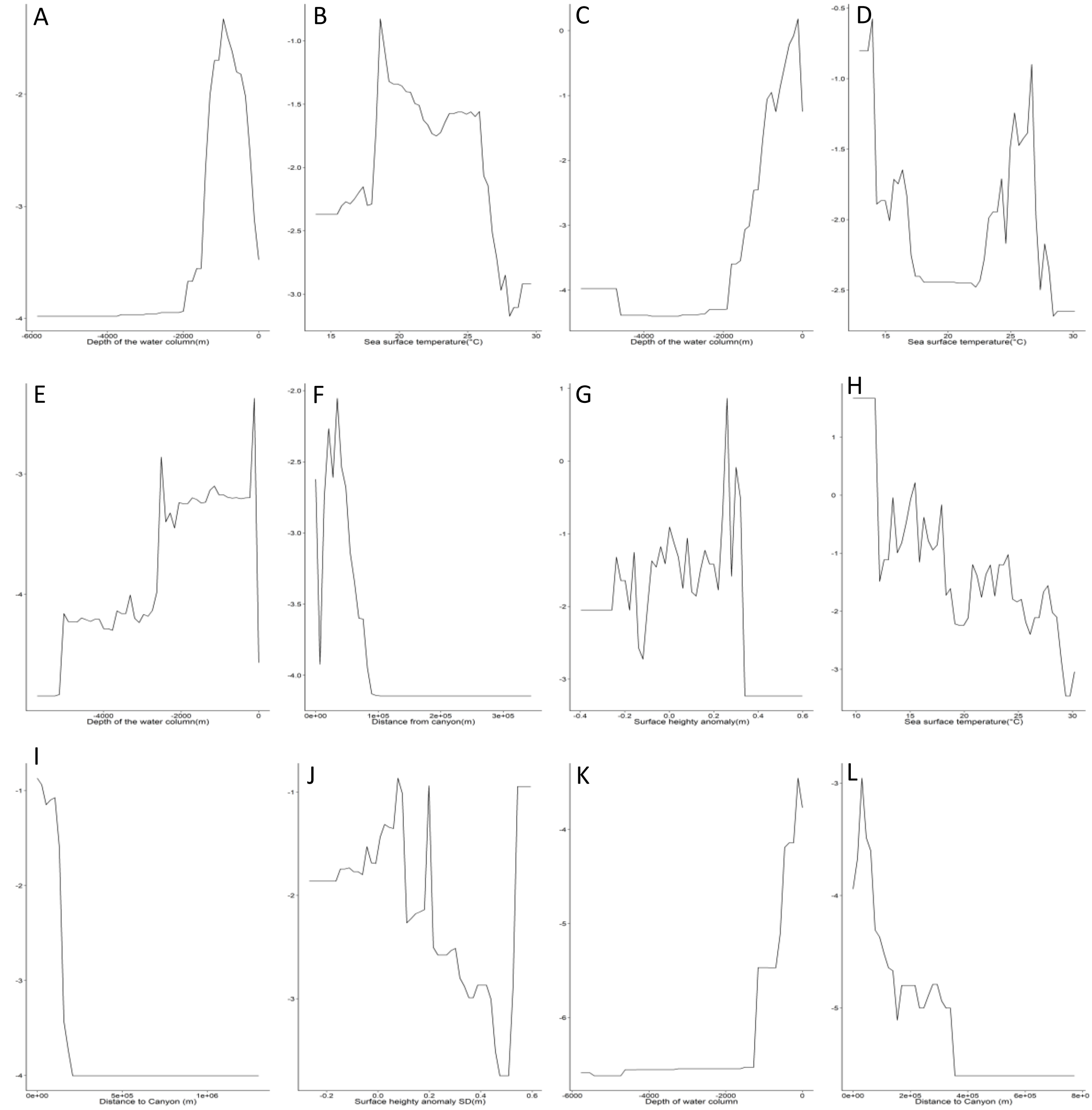

Figure S12. Partial plots showing the relationship between probability of occurrence and the top environmental variables included in final gradient boosted model containing ID as predictor for foraging in the North West (A-B), South West (C-D), southern Australia (E-F), reproduction/foraging in Southeast Asia (G-H), and for migration in western Australia (I-J) and southern Australia (K-L).

References

1. Fourcade Y, Besnard AG, Secondi J. Paintings predict the distribution of species, or the challenge of selecting environmental predictors and evaluation statistics. Global Ecol Biogeogr. 2018;27(2):245-56.
